# Differential associations of oxidative stress parameters with neuroendocrine markers and hemodynamic reactivity in acute mental stress‐induced adrenergic reactivity profiles: The SABPA study

**DOI:** 10.14814/phy2.71024

**Published:** 2026-07-21

**Authors:** Dewald Naudé, Wayne Smith, Catharina MC Mels, Roland von Känel, Annemarie Wentzel

**Affiliations:** ^1^ Hypertension in Africa Research Team North‐West University Potchefstroom South Africa; ^2^ South African Medical Research Council Unit for Hypertension and Cardiovascular Disease North‐West University Potchefstroom South Africa; ^3^ Department of Consultation‐Liaison Psychiatry and Psychosomatic Medicine University Hospital, University of Zurich Zurich Switzerland

**Keywords:** acute mental stress, alpha‐adrenergic response, beta‐adrenergic response, neuroendocrine markers, oxidative stress

## Abstract

Stress‐induced alpha (α)‐ and beta (β)‐adrenergic reactivity profiles relate to distinct neuroendocrine‐associated hemodynamic reactivity and cardiometabolic risk profiles. While oxidative stress links stress to cardiometabolic risk, its relationship with neuroendocrine markers and hemodynamics remains unclear. Within these profiles, we investigated resting oxidative stress parameters and their associations with neuroendocrine markers and hemodynamic reactivity. We included 362 participants (aged 20–65 years) and recorded beat‐to‐beat hemodynamic reactivity during the Stroop‐Color‐Word‐Conflict test. We categorized α‐responders (*n* = 47), β‐responders (*n* = 68) and mixed‐α/β‐responders (*n* = 247) based on cardiac output (∆%CO) and Windkessel arterial compliance (∆%Cwk) reactivity. Baseline fasting urine and blood samples were analyzed for neuroendocrine markers and oxidative stress parameters. Predominant α‐responders were older with higher hypertension prevalence than other responders. Oxidative stress parameters were comparable between α‐ and β‐responders. In α‐responders, SOD and GR inversely associated with ∆%CO and GPx positively with ∆%Cwk (all *p* ≤ 0.026). In β‐responders, u‐NE/Cr positively associated with GGT, ACTH inversely with ROS and tGSH positively with ∆%CO (all *p* ≤ 0.014). Our findings highlight divergence in cardiometabolic risk pathways. In α‐responders, enzymatic antioxidants may support downstream vascular hemodynamic buffering in response to high‐pressure‐related vascular risk. In β‐responders, sympathetic‐driven increases in glutathione turnover could reflect antioxidant adaptation to preserve cardiac function during acute stress.

## Graphical abstract

Summary of the Differential associations of oxidative stress parameters with neuroendocrine markers and hemodynamic reactivity in acute mental stress‐induced adrenergic reactivity profiles

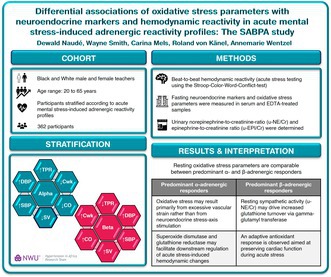

## 1 INTRODUCTION

Acute mental stress elicits hemodynamic reactivity response patterns which are involved in regulating increased cardiometabolic demands imposed by stress (Ginty et al., 2017). These hemodynamic reactivity response patterns can more accurately be classified as predominant alpha (α)‐ or beta (β)‐adrenergic reactivity patterns based on preferential α‐ or β‐adrenergic receptor activation or a combination thereof (i.e. a mixed‐α/β‐adrenergic reactivity pattern) (Gregg et al., 1999). We recently characterized these response patterns as comprehensive acute mental stress‐induced predominant α‐ and β‐adrenergic reactivity profiles with each being related to a distinct cardiometabolic risk profile (Wentzel et al., 2025). Specifically, predominant α‐adrenergic responders showed a high‐pressure‐related peripheral vascular risk profile as compared to predominant β‐adrenergic responders indicating a more metabolic‐driven risk profile along with possible hyperperfusion injury over time.

Predominant α‐ and β‐adrenergic reactivity profiles are mediated by an interplay of neuroendocrine markers associated with the sympatho‐adrenal‐medullary (SAM) system and hypothalamic–pituitary–adrenal (HPA)‐axis (McEwen, 2007; Wadsworth et al., 2019). Recently, unique associations between resting neuroendocrine markers and individual hemodynamic reactivity parameters were reported in each adrenergic reactivity profile. In predominant α‐adrenergic responders, urinary norepinephrine‐to‐creatinine ratio (u‐NE/Cr) may reflect an excessive peripheral sympathetic nervous system (SNS)‐driven response favoring vasoconstriction while adrenocorticotropic hormone (ACTH) and cortisol may facilitate decreases in cardiac output reactivity (∆%CO) and Windkessel arterial compliance reactivity (∆%Cwk) despite observed increases in total peripheral resistance reactivity (∆%TPR) (Naudé et al., 2026). In contrast, in predominant β‐adrenergic responders, a more central cardiac SNS‐driven response is evident where u‐NE/Cr and urinary epinephrine‐to‐creatinine ratio (u‐EPI/Cr) may contribute to enhanced cardiac contractility while u‐EPI/Cr may increase ∆%Cwk in an attempt to reduce vascular resistance during stress (Naudé et al., 2026). These neuroendocrine associations are significant in linking each adrenergic reactivity profile to their distinct cardiometabolic risk profiles.

Oxidative stress is a hallmark feature of initial cardiometabolic pathophysiology (Montezano et al., 2015; Touyz et al., 2020; Touyz & Briones, 2011) and has emerged as a key molecular mechanism linking stress exposure to cardiometabolic risk (Sher et al., 2020; Siegrist & Sies, 2017). Yet, few studies have examined the role of oxidative stress in acute mental stress‐induced adrenergic reactivity responses. Oxidative stress is intricately linked to neuroendocrine alterations which may reveal signs of cardiometabolic pathophysiology (Adameova et al., 2009; Costa et al., 2011). However, it is important to note that interactions between neuroendocrine markers and oxidative stress parameters within acute stress‐induced adrenergic reactivity profiles may vary across a continuum of physiological expression during exposure to an acute stressor. Indeed, catecholamines have been shown in experimental studies to exhibit antioxidant properties by chelating metal ions under normal physiological conditions which could inhibit oxidative reactions (Álvarez‐Diduk & Galano, 2015). However, in contrast, if released in excess, catecholamines may undergo auto‐oxidation to form adrenochromes and oxyradicals, which may increase oxidative stress and induce cardiotoxic effects (Adameova et al., 2009; Dhalla et al., 2010). Excess glucocorticoid levels are also linked to reactive oxygen species (ROS) overproduction in vitro and decreased nitric oxide (NO) bioavailability which could impair vasodilation of vascular smooth muscle cells in vivo (Iuchi et al., 2003). It is therefore important to explore the relationships between oxidative stress parameters, neuroendocrine markers and individual hemodynamic reactivity parameters and to determine whether such relationships are associated with a predominant adrenergic reactivity profile under acute mental stress conditions. It is evident that existing literature tends to examine these components in isolation rather than as parts of an integrated stress response system. Framing these variables as interconnected components rather than independent factors may help contextualize how distinct acute adrenergic response patterns relate to physiological processes relevant to cardiometabolic risk, without implying chronic or long‐term adaptations, while also recognizing that hemodynamic reactivity is mediated by neuroendocrine and oxidative pathways.

While not investigating hemodynamic reactivity parameters per se, Mokhaneli et al. (2016) demonstrated that thiobarbituric acid reactive substances (TBARS), a marker of oxidative damage, are associated with decreases in Windkessel arterial compliance (Cwk) and increases in total peripheral resistance (TPR) (Mokhaneli et al., 2016) which are hallmark hemodynamic characteristics of a predominant α‐adrenergic reactivity profile (Wentzel et al., 2019). Other studies indicated that altered shear stress patterns, which might be at play in predominant β‐adrenergic responders over time (Wentzel et al., 2025), may generate excess ROS via vascular oxidases (Harrison et al., 2006); thus, enhancing oxidative stress. Another study conducted in two age‐ and ethnicity‐stratified South African cohorts has previously reported associations between resting oxidative stress parameters and individual hemodynamic reactivity parameters (Myburgh et al., 2019). Specifically, hemodynamic reactivity parameters associated with gamma‐glutamyl transferase (GGT) and glutathione peroxidase (GPx) in Black South Africans and with ROS, superoxide dismutase (SOD), and total glutathione (tGSH) in White South Africans. These findings further suggest differing underlying oxidative stress‐related mechanisms of cardiovascular regulation during acute mental stress responses.

We aimed to determine how oxidative stress could be related to neuroendocrine‐linked adrenergic reactivity profiles as this could ultimately inform targeted interventions to enhance stress resilience and cardiovascular health. An oxidative stress‐focused approach may yield novel insights into the early dynamic pathophysiological processes underlying predominant α‐ and β‐adrenergic signaling during acute stress exposure beyond those explained by conventional cardiometabolic risk factors (Corbi et al., 2013; Underwood et al., 2024; Ziolkowski & Grover, 2010). In the absence of previous human studies directly examining the interplay between oxidative stress parameters, neuroendocrine markers, and hemodynamic reactivity parameters in an acute stress context, the findings of this study should be considered exploratory and hypothesis‐generating. Therefore, we compared (1) resting levels of oxidative stress parameters [ROS, tGSH, GPx, glutathione reductase (GR), SOD, and GGT] and serum NO metabolites within predominant α‐, predominant β‐, and mixed‐α/β‐adrenergic responders, and assessed (2) associations of oxidative stress parameters, neuroendocrine markers, and hemodynamic reactivity parameters in each adrenergic responder group.

## 2 MATERIALS AND METHODS

### 2.1 Study design and population

The current investigation is nested within the Sympathetic activity and Ambulatory Blood Pressure in Africans (SABPA) study and includes the baseline sample of urban‐dwelling Black and White South African male and female teachers (aged between 20 and 65 years) (*N* = 409). Detailed information pertaining to the larger study has been previously published (Malan et al., 2015). In summary, participants were recruited on a voluntary basis from the Dr. Kenneth Kaunda education distinct in the North West province of South Africa. They had similar socioeconomic and educational status, similar access to healthcare and were from a similar working environment. However, cultural differences could not be accounted for. Participants were excluded at baseline based on the following criteria: usage of α‐ or β‐blockers, pregnancy or lactation, usage of psychotropic substances, vaccinations or blood donations within the previous 3 months and tympanic temperature above 37°C. Participants with missing data for hemodynamic reactivity parameters obtained from acute mental stress testing, neuroendocrine markers, oxidative stress parameters as well as serum NO metabolites were additionally excluded (*n* = 47). The final study sample included 362 participants which were stratified according to predominant adrenergic reactivity profiles as published elsewhere (Naudé et al., 2026; Wentzel et al., 2025) (please also refer to Section 2.7).

### 2.2 Ethical considerations

The research team obtained permission from the North West Department of Education and the South African Democratic Teacher Union to conduct the larger SABPA study. The study was also approved by the Health Research Ethics Committee of North‐West University (NWU‐00036‐07‐A6). All procedures have adhered to good clinical practice and to the principles outlined in the Declaration of Helsinki of 1975 (revised 2008). The current affiliated study also adhered to the updated South African Department of Health's guidelines on Ethics in Health Research in human participants (2024). Written informed consent was obtained from all the participants prior to the commencement of any measurements. The general procedure relating to data collection has also been published elsewhere (Malan et al., 2015).

### 2.3 Questionnaire data

Demographic and general health questionnaires were completed in a private clinical research environment within the Metabolic Unit research facility at North‐West University. These questionnaires provided information pertaining to participants' age, sex, ethnicity, medication usage, medical history, family history, and lifestyle factors (including self‐reported smoking and alcohol consumption).

### 2.4 Anthropometric measurements

Body height (Invicta Stadiometer; IP1465, Invicta Plastics Ltd., Leicester, UK), body weight (Precision Health Scale; A&D Company, Tokyo, Japan) and waist circumference (WC) (Anthropometric tape; Holtain, Croswell, Wales) were measured in triplicate by registered level II anthropometrists according to standardized methods of the International Society for the Advancement of Kinanthropometry (Marfell‐Jones et al., 2012). Subsequently, body mass index (BMI) (body weight [kg]/square of body height [m^2^]) was calculated.

### 2.5 Ambulatory blood pressure measurements

A suitable cuff was fitted to the non‐dominant arm of each participant early on the first day of data collection. 24‐h ambulatory systolic (SBP) and diastolic (DBP) blood pressure were measured in 30‐min intervals during the day (08:00 to 22:00) and every hour during nighttime (22:00 to 06:00) by use of the Cardiotens CE120® (Meditech, Budapest, Hungary). Participants were requested to record any abnormalities such as headaches or nausea on their issued diary cards for the whole duration of the measurement. The ABPM device was then removed from the participant early on the next morning and CardioVisions 1.19 Personal Edition Software (Meditech®) was subsequently used to analyze the data. The 24‐h mean arterial pressure (MAP) was calculated as 23DBP+13SBP. We applied the 2020 International Society of Hypertension (ISH) global hypertension practice guidelines (Unger et al., 2020) to define 24‐h hypertensive status as 24‐h ABPM SBP ≥130 mmHg and/or 24‐h ABPM DBP ≥80 mmHg and/or being on anti‐hypertensive medication. The ISH guidelines were used in this study as these guidelines were the first to be specifically developed for hypertension management in all regions of the world, regardless of population or resources.

### 2.6 Hemodynamic reactivity

Beat‐to‐beat hemodynamic reactivity was continuously and non‐invasively measured using the validated Finometer device (Finapres Medical Systems®, Amsterdam, the Netherlands) (Guelen et al., 2008; Schutte et al., 2003; Schutte et al., 2004). Following 30 min of rest in a semi‐recumbent position, each participant underwent a 5‐min baseline beat‐to‐beat recording of their blood pressure (BP). A return‐to‐flow systolic calibration was performed after the first 2 min to calibrate finger arterial pressure against brachial artery pressure for each participant, thereby ensuring precise measurements in accordance with the standards of the Association for the Advancement of Medical Instrumentation. An additional 5–10 min of rest was provided to ensure hemodynamic stabilization before administration of the acute mental stress task, namely the Stroop‐Color‐Word‐Conflict (Stroop‐CWC) test. This acute mental stress task was used to elicit acute mental stress‐induced hemodynamic reactivity responses (Stroop, 1935) as it evokes physiological changes characteristic of sympathoadrenal activation (Tulen et al., 1989). The Stroop‐CWC test was administered for 1 min and has previously demonstrated reproducibility in cardiovascular reactivity over both a 2‐h (Freyschuss et al., 1988) and a 1‐month (Fauvel et al., 1996) period. During the administration of this stress task, participants were required to identify the ink color of printed words while inhibiting their natural inclination to read the printed word (e.g., the word “GREEN” printed in purple ink). Acute mental stress testing procedures were conducted by an independent observer who maintained a neutral demeanor while correcting incorrect responses and encouraging faster performance. Each participant received a performance‐based monetary incentive upon the completion of the stress task which encouraged active engagement of the participant and ensured that an accurate physiological stress response was recorded. True baseline values were taken as the average of the last 3 min of the resting recordings, whereas stress reactivity values were derived from the average of the last 20–30 s of the stressor recordings. Finometer data were further processed using Beat‐Scope version 1.1.a software (Finapres Measurement Systems) to derive hemodynamic parameters including SBP, DBP, cardiac output (CO), stroke volume (SV), heart rate (HR), TPR and Cwk. Maximal reactivity of each hemodynamic parameter was calculated as the percentage change from baseline using the following formula: Reactivity (∆%) = [(X_stressor_ – X_baseline_)/X_baseline_] × 100 (Cinciripini, 1986).

### 2.7 Population stratification

Differential hemodynamic response patterns based on either predominant α‐ or β‐adrenergic receptor activation are elicited during acute mental stress exposure (Gregg et al., 1999). It is well‐established that predominant α‐adrenergic receptor activation during acute stress exposure hemodynamically translates to a reactivity pattern characterized by higher TPR and DBP with concomitant decreases in CO and Cwk (Kasprowicz et al., 1990; Sherwood & Turner, 1995; van Rooyen et al., 2002; Wentzel et al., 2019). On the other hand, predominant β‐adrenergic receptor activation during acute stress exposure elicits a hemodynamic reactivity pattern consistent with elevated CO and Cwk with lower TPR (Kasprowicz et al., 1990; Sherwood & Turner, 1995; van Rooyen et al., 2002; Wentzel et al., 2019). In line with our work published elsewhere (Naudé et al., 2026; Wentzel et al., 2025), participants were stratified into dichotomous α‐ and β‐adrenergic responder groups using a quartile‐based stratification method involving reactivity of both cardiac output (∆%CO) and Windkessel arterial compliance (∆%Cwk). Participants in the lowest quartile group of both ∆%CO and ∆%Cwk were classified as predominant α‐adrenergic responders (*n* = 47), while participants in the highest quartile group of both ∆%CO and ∆%Cwk were classified as predominant β‐adrenergic responders (*n* = 68). All remaining participants were classified as mixed‐α/β‐adrenergic responders (*n* = 247). Reactivity of CO and Cwk were selected as the primary variables used for adrenergic profiling because of their joint hemodynamic relevance. Indeed, CO, which is determined as the product of HR and SV (Frank, 1895), is a major determinant of SBP (Vest, 2019), whereas Cwk reflects the capacity of small and large arteries to distend and recoil during the cardiac cycle (Brar, 2016). This is consistent with the classic two‐element Windkessel model which characterizes arterial hemodynamics through the interaction between arterial compliance and vascular resistance (Frank, 1895; Frank, 1899) as well as Poiseuille's law (Westerhof et al., 2009). Furthermore, the combined use of these two parameters (i.e., ∆%CO and ∆%Cwk) reduces the risk of statistical overadjustment and collinearity of interrelated hemodynamic parameters while providing a more comprehensive representation of the distinct hemodynamic patterns associated with adrenergic predominance during stress exposure, especially where distinct differences in adrenergic reactivity phenotypes are observed (i.e., in α‐responders: low CO/Cwk; high TPR, in β‐responders: high CO/Cwk; low TPR).

### 2.8 Biological sampling and biochemical analyses

A registered research nurse collected baseline fasting blood and urine samples from participants in the early morning (before 08:00) prior to the administration of the Stroop‐CWC test. These samples therefore represent pre‐stressor baseline levels of oxidative stress parameters, neuroendocrine and inflammatory markers and do not reflect acute responses to the mental stress task. All biological samples were immediately processed, aliquoted into cryovials, and stored at −80°C until analysis. Urinary catecholamines (norepinephrine [NE] and epinephrine [EPI]) were analyzed from acidified samples taken from the eight‐hour urine collection using a 3‐Cat Urine ELISA Fast Track kit (LDN, Nordhorn, Germany) (Catalogue number: BA E‐6600R), and were corrected for urinary creatinine, and reported as u‐NE/Cr and u‐EPI/Cr in the results section (Malan et al., 2016). Urine for catecholamine analysis was collected in containers acidified with hydrochloric acid to prevent catecholamine breakdown (Maclagan, 2012) and kept on ice or refrigerated until aliquoting. All urine samples were collected from participants early on the second morning of data collection (Malan et al., 2015). Notably, catecholamine measurements from 8‐ or 12‐h urine collections are comparable to those from the preferred 24‐h collections (Reuben et al., 2000).

Resting (pre‐stress) serum ACTH and cortisol samples were analyzed by the e411 (Roche, Basel, Switzerland). Serum lipids (total cholesterol, high‐density lipoprotein cholesterol (HDL‐C), and triglycerides), ultra‐high sensitivity serum C‐reactive protein (CRP), serum GGT and fasting blood glucose (in sodium fluoride samples) were analyzed with the UniCel DXC 800 (Beckman & Coulter, Germany) and the Konelab™ 20I Sequential Multiple Analyzer Computer (ThermoScientific, Vantaa, Finland). Serum insulin was analyzed using the Elecsys 2010 (Roche, Basel, Switzerland). Thereafter, low‐density lipoprotein cholesterol (LDL‐C) (Fredrickson et al., 1972), cholesterol‐to‐HDL‐C ratio (Chol/HDL‐C), and the homeostatic model assessment for insulin resistance (HOMA‐IR) (Matthews et al., 1985) were calculated.

Serum and urinary creatinine along with glycated hemoglobin (HbA1c) (in ethylenediaminetetraacetic acid (EDTA) whole blood samples) were measured using the Cobas® Integra 400 plus (Roche, Basel, Switzerland). Thereafter, we determined abnormal glucose tolerance (Abnl‐GT), which is a term that combines prediabetes and diabetes (Ishimwe et al., 2021), as HbA1c ≥5.7% and/or fasting plasma glucose ≥5.6 mmol/L and/or being on anti‐diabetic medication (American Diabetes Association, 2019). The estimated glomerular filtration rate (eGFR) was calculated using the 2021 Chronic Kidney Disease Epidemiology Collaboration Creatinine, Age and Sex Equation (Inker et al., 2021). Serum tumor necrosis factor‐alpha (TNF‐α) and plasma interleukin‐6 (IL‐6) values were derived from Quantikine High‐Sensitivity Human TNF‐α and IL‐6 enzyme‐linked immunosorbent assays (HS ELISA; R&D Systems, Minneapolis, MN USA) (Catalogue numbers HSTA00D and HS600C), respectively. Cotinine was analyzed in serum samples through homogenous immunoassay with an automated Modular system (Roche, Switzerland).

Resting SOD (in serum), GR (in EDTA plasma) and GPx (in EDTA plasma) were measured in duplicate using assay kits with item numbers 706,002, 703,202 and 703,102 (Cayman Chemical Company, Ann Arbor, MI, USA) respectively. All assays were performed on a Synergy H4 hybrid microplate reader (BioTek, Winooski, VT, USA). Total glutathione (in EDTA whole blood) was measured at rest using BIOXYTECH GSH/GSSG‐412 kits from OXIS research (Portland, OR) which were analyzed on the BioTek FL600 microplate fluorescence reader. Reactive oxygen species were measured as serum peroxides with the method described by Hayashi et al. (2007) on a Synergy HT microplate reader (BioTek, Winooski, VT, USA) and are reported in units, where 1 mg hydrogen peroxide per liter (H_2_O_2_/L) equates to 1 unit (Hayashi et al., 2007). The resting concentration of serum NO metabolites, the sum of plasma nitrite and reduced nitrate, was determined with an R&D Systems Inc. kit (Parameter™, Catalogue number: KGE001, MN, USA), Universal ELX800 plate reader and GEN5TM software (BioTek Instruments Inc., Winooski, VT, USA). Proteins were removed before analysis by ultrafiltration using 10,000 molecular weight cut‐off filters.

### 2.9 Statistical analyses

Statistical analyses were performed using IBM® SPSS® Statistics version 30 software (IBM Corporation; Armonk, New York, USA). All variables were assessed for normality by means of quantile‐quantile plots (visual inspection) along with both the Kolmogorov–Smirnov and Shapiro–Wilk tests (p‐value inspection). The Kolmogorov–Smirnov test was applied to the larger mixed‐α/β‐adrenergic responder group, while the Shapiro–Wilk test was used for the smaller predominant α‐ and β‐adrenergic responder groups, as it is more suitable for sample sizes below 50. Box‐Cox logarithmic transformations (Box & Cox, 1964) were conducted for oxidative stress parameters (i.e. ROS, tGSH, GPx, GR, GGT and SOD) and NO metabolites, yielding near‐normal distributions superior to those from natural or base‐10 logarithmic transformations. All other non‐parametric data were logarithmically transformed using the natural logarithm. Individual hemodynamic reactivity parameters (∆%SBP, ∆%DBP, ∆%TPR, ∆%CO, ∆%HR, ∆%SV, ∆%Cwk) were not logarithmically transformed as these variables are expressions of percentage change from baseline.

Basic characteristics of the sample were determined using analyses of covariance (ANCOVA) (adjustments applied for age, sex, and ethnicity) and Chi‐square tests. Chronological age was compared between the different adrenergic responder groups using Welch's analyses of variance (ANOVA). All hemodynamic reactivity parameters were compared between adrenergic responder groups using ANCOVA, adjusting for age, sex, ethnicity, WC, Abnl‐GT, hypertensive status, self‐reported smoking, and alcohol use to account for conventional cardiometabolic risk factors. Given that previous research has demonstrated that hemodynamic reactivity responses may vary as a function of age, sex, and/or ethnicity (Anderson, 1989; Hastrup & Light, 1984; Huisman, 1997), we consistently adjusted for these demographic factors in all of our previous (Naudé et al., 2026; Wentzel et al., 2025) and present analyses. This strategy minimizes the influence of non‐modifiable determinants of hemodynamic stress reactivity responses, thereby facilitating the non‐invasive characterization of predominant α‐ and β‐adrenergic reactivity phenotypes based on underlying differences in hemodynamic reactivity patterns. Resting neuroendocrine markers and oxidative stress parameters were compared between adrenergic reactivity profiles using unadjusted Kruskal–Wallis tests and adjusted ANCOVA. Adjustments included age, sex, ethnicity, self‐reported smoking status, self‐reported alcohol use, anti‐inflammatory medication use, antioxidant and multivitamin intake, as well as oral contraceptive use and female hormonal therapy to control for non‐modifiable risk, lifestyle, medication, and supplement‐related confounding. A two‐tailed *p*‐value <0.050 was considered statistically significant.

The most physiologically justified and statistically relevant confounders for backward stepwise multiple regression analyses were chosen based on exploratory Spearman rank correlations between resting oxidative stress parameters (ROS, tGSH, GPx, GR, GGT, SOD) and serum NO metabolites as dependent variables and various other markers (age, sex, ethnicity, cotinine, self‐reported smoking status, self‐reported alcohol use, body composition markers, 24‐h BP, glucose metabolism markers, inflammatory markers, lipid profile, eGFR, and various medication classes) as independent variables (Tables S3–S9). The final confounders used in multiple regression analyses included age, sex, ethnicity, self‐reported smoking status, self‐reported alcohol use, anti‐inflammatory medication use, antioxidant and multivitamin intake, oral contraceptive use, and female hormonal therapy.

Backward stepwise multiple regression analyses were used to identify independent associations in several models between the main markers as delineated below:

*Model type 1 (Associations between oxidative stress and neuroendocrine markers)*: Each model included one oxidative stress marker as the dependent variable, one neuroendocrine marker as the independent variable, and the abovementioned list of confounders.

*Model type 2 (Associations between oxidative stress parameters and hemodynamic reactivity parameters)*: Each model included one hemodynamic reactivity parameter as a dependent variable, one oxidative stress marker as an independent variable, and the abovementioned list of confounders.

This study was conducted from an observational and physiological perspective to explore associations between the primary markers of interest within an integrated acute stress response system which justifies the use of separate regression models as described above. Specifically, we examined how neuroendocrine activation relates to oxidative stress parameters, and how these, in turn, associate with individual hemodynamic reactivity parameters within each adrenergic responder group, acknowledging that hemodynamic responses are downstream manifestations of neuroendocrine and oxidative processes rather than independent factors. These analyses were intended to identify potential physiological linkages between these interconnected systems during acute stress exposure, rather than to infer sustained or causal mechanisms. The results should therefore be interpreted as exploratory and hypothesis‐generating. We did not correct for multiple hypothesis testing, as this approach was used to avoid multicollinearity among interrelated variables in the regression models.

## 3 RESULTS

### 3.1 Baseline characteristics of participants

Baseline characteristics are reported in Table 1. Predominant α‐adrenergic responders were older, included more participants of Black ethnicity, had higher 24‐h BP values, and had a higher prevalence of hypertension and Abnl‐GT compared to predominant β‐adrenergic and mixed‐α/β‐adrenergic responders (all *p* ≤ 0.013). Independent of age, sex, ethnicity, WC, Abnl‐GT, hypertensive status, self‐reported smoking and alcohol use, we observed in predominant α‐adrenergic responders, greater increases in ∆%BP and ∆%TPR together with a smaller elevation in ∆%HR and greater decreases in ∆%SV, ∆%CO and ∆%Cwk compared to predominant β‐adrenergic and mixed‐α/β‐adrenergic responders (all *p* ≤ 0.041). The medical history and medication usage of all participants are additionally reported in Table S1.

**TABLE 1:** Baseline characteristics of participants stratified by acute mental stress‐induced adrenergic reactivity profiles (*N* = 362).

	α‐Adrenergic reactivity profile (*n* = 47)	Mixed‐α/β‐adrenergic reactivity profile (*n* = 247)	β‐Adrenergic reactivity profile (*n* = 68)	*p*
*Unadjusted analyses*				
Demographic and lifestyle information				
Age, years^†^	48.9 ± 7.71^ac^	44.8 ± 9.47^ab^	41.2 ± 11.0^bc^	**<0.001**
Sex, male, *n* (%)	21 (44.7)	128 (51.8)	29 (42.6)	0.33
Ethnicity, Black, *n* (%)	30 (63.8)	116 (47.0)	17 (25.0)	**<0.001**
Self‐reported smoking, yes, *n* (%)	7 (14.9)	32 (13.0)	13 (19.1)	0.44
Self‐reported alcohol use, yes, *n* (%)	14 (29.8)	96 (39.0)	26 (38.2)	0.49
Adjusted analyses*				
Body composition				
Body mass index, kg/m^2^	28.5 (26.6; 30.4)	29.5 (28.7; 30.4)^b^	27.2 (25.6; 28.8)^b^	**0.035**
Waist circumference, cm	92.8 (88.3; 97.2)	94.5 (92.6; 96.4)	90.7 (87.0; 94.4)	0.19
24‐h ambulatory blood pressure				
Systolic blood pressure, mmHg	134 (130; 138)^ac^	128 (126; 130)^a^	124 (121; 128)^c^	**0.002**
Diastolic blood pressure, mmHg	82 (80; 85)	80 (78; 81)	78 (76; 80)	0.061
Mean arterial pressure, mmHg	99 (96; 102)^c^	96 (94; 97)	93 (91; 96)^c^	0.013
Hypertensive status, yes, *n (%)*	36 (76.6)	135 (54.7)	22 (32.4)	**<0.001**
Hemodynamic reactivity (∆%)^‡^				
Systolic blood pressure	15.8 (12.6; 18.9)^c^	13.7 (12.3; 15.0)	10.5 (7.85; 13.2)^c^	**0.041**
Diastolic blood pressure	23.5 (20.6; 26.4)^ac^	13.7 (12.4; 14.9)^ab^	4.89 (2.41; 7.37)^bc^	**<0.001**
Total peripheral resistance	68.8 (52.3; 85.3)^ac^	−1.24 (−8.26; 5.78)^a^	−20.2 (−34.2; −6.26)^c^	**<0.001**
Heart rate	22.4 (16.4; 28.4)^ac^	31.9 (29.3; 34.4)^a^	36.0 (31.0; 41.1)^c^	**0.003**
Stroke volume	−22.8 (−27.0; −18.6)^ac^	−6.27 (−8.07; −4.47)^ab^	9.04 (5.46; 12.6)^ac^	**<0.001**
Cardiac output	−5.17 (−12.7; 2.38)^ac^	23.3 (20.1; 26.5)^ab^	48.7 (42.3; 55.1)^bc^	**<0.001**
Windkessel arterial compliance	−26.9 (−30.2; −23.6)^ac^	−15.9 (−17.3; −14.5)^ab^	−3.19 (−6.01; −0.38)^bc^	**<0.001**
Biochemical analyses				
HbA1c, %	5.86 (5.66; 6.07)	5.72 (5.63; 5.80)	5.60 (5.44; 5.77)	0.17
Glucose, mmol/L	5.67 (5.35; 6.01)	5.58 (5.44; 5.72)	5.34 (5.09; 5.61)	0.23
Insulin, μU/mL	11.2 (9.48; 13.2)	11.1 (10.3; 11.9)	9.82 (8.55; 11.3)	0.31
HOMA‐IR, %	2.82 (2.33; 3.41)	2.75 (2.53; 2.98)	2.33 (1.99; 2.73)	0.18
Abnl‐GT, yes, *n (%)*	33 (70.2)	161 (65.2)	24 (35.3)	**<0.001**
C‐reactive protein, mg/L	3.41 (2.55; 4.57)	2.86 (2.53; 3.24)	2.59 (2.04; 3.30)	0.39
Interleukin‐6, pg/mL	1.03 (0.94; 1.12)	1.00 (0.97; 1.04)	1.00 (0.93; 1.08)	0.87
Tumor necrosis factor‐alpha, pg/mL	1.31 (0.85; 1.99)	1.16 (0.97; 1.40)	0.94 (0.66; 1.34)	0.47
Total cholesterol, mmol/L	5.08 (4.72; 5.44)	5.13 (4.98; 5.28)	5.28 (4.98; 5.57)	0.65
HDL‐cholesterol, mmol/L	1.14 (1.04; 1.24)	1.16 (1.12; 1.20)	1.23 (1.15; 1.31)	0.29
LDL‐cholesterol, mmol/L	3.67 (3.35; 3.98)	3.71 (3.58; 3.85)	3.79 (3.53; 4.05)	0.84
Triglycerides, mmol/L	1.19 (1.02; 1.39)	1.07 (1.07; 1.14)	0.98 (0.86; 1.12)	0.17
Cholesterol‐to‐HDL ratio	4.56 (4.16; 4.99)	4.51 (4.34; 4.69)	4.27 (3.96; 4.60)	0.41
eGFR, mL/min/1.73 m^2^	99.0 (95.0; 103)	99.4 (97.6; 101)	97.8 (94.3; 101)	0.72
Cotinine, ng/mL	0.062 (0.022; 0.17)	0.051 (0.033; 0.079)	0.062 (0.027; 0.15)	0.90

*Note*: Data expressed as arithmetic mean ± standard deviation, arithmetic mean (95% confidence intervals), geometric mean (95% confidence intervals) for logarithmically transformed variables or frequency and percentage of participants (*n, %*). Bold values denote *p* < 0.050. All *p*‐values obtained via Chi‐square tests, ^†^Welch's ANOVA, ANCOVA with adjustments applied for *age, sex, and ethnicity or ^‡^age, sex, ethnicity, waist circumference, hypertensive status, Abnl‐GT, self‐reported smoking, and alcohol use. Hypertensive status is defined as 24‐h ambulatory blood pressure ≥130/80 mmHg and/or being on anti‐hypertensive medication. Abnl‐GT is defined as HbA1c ≥5.7% and/or fasting plasma glucose ≥5.6 mmol/L and/or being on anti‐diabetic medication. Significant differences between ^a^α‐adrenergic responders and mixed‐α/β‐adrenergic responders, ^b^β‐adrenergic responders and mixed‐α/β‐adrenergic responders and ^c^α‐adrenergic responders and β‐adrenergic responders were obtained with Games‐Howell and Dunn‐Bonferroni post‐hoc tests.

Abbreviations: ∆%, reactivity; Abnl‐GT, abnormal glucose tolerance; eGFR, estimated glomerular filtration rate; HbA1c, glycated hemoglobin; HDL, high‐density lipoprotein; HOMA‐IR, homeostatic model assessment for insulin resistance; LDL, low‐density lipoprotein.

In both unadjusted and adjusted analyses, there were no significant group differences in neuroendocrine markers (u‐NE/Cr, u‐EPI/Cr, ACTH, and cortisol) among the three groups (Table S2 and Table 2). In unadjusted analyses (Table S2), predominant α‐adrenergic responders had lower GPx and higher ROS, GR, GGT, and NO metabolite levels compared to predominant β‐adrenergic and mixed‐α/β‐adrenergic responders (all *p* ≤ 0.012). These findings were notably absent after adjusting for age, sex, and ethnicity, and remained absent after further adjustment for self‐reported smoking status, self‐reported alcohol use, anti‐inflammatory medication use, antioxidant and multivitamin intake, oral contraceptive use, and female hormonal therapy (Table 2).

**TABLE 2:** Adjusted comparisons of neuroendocrine markers and oxidative stress parameters in groups stratified according to acute mental stress‐induced adrenergic reactivity profiles (*N* = 362).

	α‐adrenergic reactivity profile (*n* = 47)	Mixed‐α/β‐adrenergic reactivity profile (*n* = 247)	β‐adrenergic reactivity profile (*n* = 68)	*p*
Neuroendocrine markers				
Adrenocorticotropic hormone, pg/mL	16.5 (14.0; 19.4)	15.9 (14.9; 17.1)	14.9 (13.0; 17.1)	0.61
Cortisol, nmol/L	309 (268; 357)	348 (328; 370)	331 (294; 373)	0.29
u‐NE/Cr, nmol/mmol	18.9 (14.9; 23.9)	19.2 (17.4; 21.2)	17.9 (14.7; 21.9)	0.83
u‐EPI/Cr, nmol/mmol	2.41 (1.90; 3.05)	2.72 (2.46; 3.00)	2.67 (2.19; 3.26)	0.66
Oxidative stress parameters				
^†^Reactive oxygen species, units	193 (154; 231)	159 (127; 199)	146 (118; 180)	0.17
^†^Total glutathione, μM	800 (717; 936)	871 (741; 970)	828 (714; 1007)	0.28
^†^Glutathione peroxidase, nmol/min/mL	30.6 (22.4; 38.7)	33.4 (28.5; 42.3)	35.9 (29.8; 40.9)	0.051
^†^Glutathione reductase, nmol/min/mL	6.37 (3.82; 10.2)	4.84 (2.80; 7.64)	4.33 (2.61; 6.56)	0.32
^†^Superoxide dismutase, U/mL	4.35 (3.52; 6.68)	4.15 (2.88; 5.81)	4.51 (2.97; 5.94)	0.35
^†^Gamma‐glutamyl transferase, U/L	33.0 (25.0; 71.7)	27.6 (18.0; 50.0)	17.5 (12.3; 30.6)	0.075
^†^Nitric oxide metabolites, μmol/L	4.88 (1.25; 9.18)	2.47 (0.67; 9.23)	1.51 (0.53; 4.57)	1.00

*Note*: Data expressed as geometric mean (95% confidence intervals) for neuroendocrine markers transformed to the natural logarithm or ^†^median (25th and 75th percentiles) for Box‐Cox transformed oxidative stress parameters. All *p*‐values obtained via analyses of covariance (ANCOVA). Adjustments were applied for age, sex, ethnicity, self‐reported smoking status, self‐reported alcohol usage, anti‐inflammatory medication usage, hormone replacement therapy, female oral contraception use, antioxidant intake, and multivitamin intake. Reactive oxygen species measured as serum peroxides where 1 unit = 1.0 mg/L H_2_O_2_. Nitric oxide metabolites measured as the sum of plasma nitrite and reduced nitrate.

### 3.2 Adjusted associations

Table 3 only reflects the significant associations between neuroendocrine markers and oxidative stress parameters in each adrenergic reactivity profile. Similarly, Table 4 indicates only the significant associations between oxidative stress parameters and hemodynamic reactivity parameters in each adrenergic reactivity profile. No significant associations between neuroendocrine markers and oxidative stress parameters were observed in the predominant α‐adrenergic responder group. However, SOD and GR were inversely associated with ∆%CO (all *p* ≤ 0.026) while GPx was positively associated with ∆%Cwk (*p* = 0.019) in the same responder group. Yet, in predominant β‐adrenergic responders, the u‐NE/Cr was positively associated with GGT (*p* = 0.013) while ACTH was inversely associated with ROS (*p* = 0.008). In the same group, tGSH was positively associated with ∆%CO (*p* = 0.014).

**TABLE 3:** Backward multiple regression analyses of neuroendocrine markers and oxidative stress parameters stratified according to acute mental stress‐induced adrenergic reactivity profiles (*N* = 362).

	Predominant α‐adrenergic reactivity profile (*n* = 47)		Mixed‐α/β‐adrenergic reactivity profile (*n* = 247)		Predominant β‐adrenergic reactivity profile (*n* = 68)	
	*ß* (±95% CI)	*p*	*ß* (±95% CI)	*p*	*ß* (±95% CI)	*p*
*Dependent variable: Reactive oxygen species*						
Adj *R* ^2^	0.48		0.33		0.24	
ACTH	NS	NS	NS	NS	−35.0 (−60.5; −9.53)	**0.008**
Sex	−51.1 (−73.9; −28.4)	**<0.001**	−62.4 (−76.6; −48.1)	**<0.001**	−42.5 (−69.0; −16.0)	**0.002**
Ethnicity	−61.9 (−85.3; −38.5)	**<0.001**	−31.4 (−45.4; −17.5)	**<0.001**	−36.0 (−66.8; −5.22)	**0.023**
Self‐reported smoking	NS	NS	24.8 (4.53; 45.1)	**0.017**	NS	NS
Self‐reported alcohol use	NS	NS	16.5 (1.74; 31.2)	**0.029**	NS	NS
Female hormonal therapy	NS	NS	NS	NS	27.1 (2.22; 52.0)	**0.033**
Oral contraceptive use	NS	NS	40.8 (13.2; 68.5)	**0.004**	NS	NS
Anti‐inflammatory medication	29.8 (0.26; 59.3)	**0.048**	NS	NS	NS	NS
*Dependent variable: Glutathione peroxidase*						
Adj *R* ^2^	0.059		0.029		0.10	
u‐NE/Cr	NS	NS	1.73 (0.11; 3.35)	**0.036**	NS	NS
Age	NS	NS	NS	NS	0.19 (0.014; 0.37)	**0.035**
Ethnicity	7.03 (−0.17; 14.2)	0.055	2.68 (−0.18; 5.54)	0.066	NS	NS
Female hormonal therapy	NS	NS	NS	NS	3.47 (−0.15; 7.08)	0.060
Multivitamin intake	NS	NS	NS	NS	−11.4 (−23.1; 0.25)	0.055
Adj *R* ^2^	0.059		0.042		0.10	
u‐EPI/Cr	NS	NS	2.43 (0.72; 4.15)	**0.006**	NS	NS
Age	NS	NS	NS	NS	0.19 (0.014; 0.37)	**0.035**
Sex	NS	NS	NS	NS	NS	NS
Ethnicity	7.03 (−0.17; 14.2)	0.055	3.13 (0.32; 5.94)	**0.029**	NS	NS
Female hormonal therapy	NS	NS	NS	NS	3.47 (−0.15; 7.08)	0.060
Multivitamin intake	NS	NS	NS	NS	−11.4 (−23.1; 0.25)	0.055
*Dependent variable: Glutathione reductase*						
Adj *R* ^2^	0.41		0.34		0.32	
u‐NE/Cr	NS	NS	0.65 (0.087; 1.20)	**0.024**	NS	NS
Age	NS	NS	NS	NS	0.079 (0.013; 0.15)	**0.020**
Sex	NS	NS	−1.20 (−2.20; −0.19)	**0.020**	NS	NS
Ethnicity	−5.79 (−8.15; −3.44)	**<0.001**	−5.55 (−6.56; −4.54)	**<0.001**	−3.95 (−5.62; −2.28)	**<0.001**
Self‐reported smoking	2.96 (−0.22; 6.14)	0.068			NS	NS
Self‐reported alcohol use	NS	NS	1.97 (0.93; 3.02)	**<0.001**	NS	NS
Multivitamin intake	NS	NS	NS	NS	−5.66 (−9.98; −1.35)	**0.011**
*Dependent variable: Gamma‐glutamyl transferase*						
	0.60		0.41		0.51	
u‐NE/Cr	NS	NS	7.95 (0.94; 15.0)	**0.026**	20.3 (4.47; 36.0)	**0.013**
Sex	57.4 (30.7; 84.0)	**<0.001**	47.0 (34.4; 59.6)	**<0.001**	50.1 (27.4; 72.8)	**<0.001**
Ethnicity	−78.1 (−105; −51.0)	**<0.001**	−65.7 (−78.7; −52.7)	**<0.001**	−84.4 (−109; −59.9)	**<0.001**
Self‐reported alcohol use	NS	NS	18.3 (5.18; 31.4)	**0.006**	NS	NS
Oral contraceptive use	64.2 (0.19; 128)	**0.049**	NS	NS	NS	NS
Anti‐inflammatory medication	NS	NS	NS	NS	62.3 (18.0; 107)	**0.007**
Multivitamin intake	−54.8 (−118; 8.15)	0.086	−23.2 (−47.1; 0.75)	0.058	NS	NS
*Dependent variable: Nitric oxide metabolites*						
	0.37		0.32		0.21	
u‐NE/Cr	NS	NS	−1.25 (−2.45; −0.045)	**0.042**	−2.48 (−5.39; 0.43)	0.094
Sex	NS	NS	−1.93 (−4.04; 0.17)	0.072	NS	NS
Ethnicity	−8.87 (−12.4; −5.32)	**<0.001**	−10.2 (−12.3; −8.10)	**<0.001**	−9.04 (−13.9; −4.13)	**<0.001**
Anti‐inflammatory medication	NS	NS	4.21 (−0.58; 8.99)	0.085	NS	NS
Antioxidant intake	16.0 (4.17; 27.8)	**0.009**	NS	NS	NS	NS

*Note*: All models were adjusted for age, sex, ethnicity, self‐reported smoking status, self‐reported alcohol use, antioxidant and multivitamin intake, anti‐inflammatory medication usage, female hormonal therapy, and oral contraceptive usage. All models were statistically significant (all *p* ≤ 0.011) unless stated otherwise. VIF values were all ≤1.177. Bold values denote statistical significance (*p* < 0.050).

Abbreviations: ACTH, adrenocorticotropic hormone; CI, confidence interval; NS, not significant; u‐EPI/Cr, urinary epinephrine‐to‐creatinine ratio; u‐NE/Cr, urinary norepinephrine‐to‐creatinine ratio.

**TABLE 4:** Backward multiple regression analyses of oxidative stress parameters and hemodynamic reactivity parameters stratified according to acute mental stress‐induced adrenergic reactivity profiles (*N* = 362).

	Predominant α‐adrenergic reactivity profile (*n* = 47)		Mixed‐α/β‐adrenergic reactivity profile (*n* = 247)		Predominant β‐adrenergic reactivity profile (*n* = 68)	
	*ß* (±95% CI)	*p*	*ß* (±95% CI)	*p*	*ß* (±95% CI)	*p*
*Dependent variable: Cardiac output reactivity (∆%CO)*						
Adj *R* ^2^	0.17		0.040		0.037	
Reactive oxygen species	NS	NS	−0.048 (−0.093; −0.003)	**0.038**	NS	NS
Age	NS	NS	−0.37 (−0.67; −0.058)	**0.020**	NS	NS
Self‐reported smoking	NS	NS	−7.69 (−16.3; 0.94)	0.080	NS	NS
Oral contraceptive use	−25.6 (−49.4; −1.80)	**0.036**	NS	NS	NS	NS
Anti‐inflammatory medication	NS	NS	NS	NS	−35.6 (−73.1; 1.90)	0.062
Antioxidant intake	41.3 (8.02; 74.6)	**0.016**	NS	NS	NS	NS
Adj *R* ^2^	0.17		0.051		0.078	
Total glutathione	NS	NS	NS	NS	0.061 (0.013; 0.11)	**0.014**
Age	NS	NS	−0.44 (−0.75; −0.13)	**0.005**	NS	NS
Ethnicity	NS	NS	7.91 (2.11; 13.7)	**0.008**	18.2 (−2.50; 38.9)	0.084
Self‐reported smoking	NS	NS	−8.67 (−17.3; −0.079)	**0.048**	NS	NS
Oral contraceptive use	−25.6 (−49.4; −1.80)	**0.036**	NS	NS	NS	NS
Antioxidant intake	41.3 (8.02; 74.6)	**0.016**	NS	NS	NS	NS
Adj *R* ^2^	0.22		0.051		0.037	
Glutathione reductase	−1.13 (−2.12; −0.14)	**0.026**	NS	NS	NS	NS
Age	NS	NS	−0.44 (−0.75; −0.13)	**0.005**	NS	NS
Ethnicity	NS	NS	7.91 (2.11; 13.7)	**0.008**	NS	NS
Self‐reported smoking	NS	NS	−8.67 (−17.3; −0.079)	**0.048**	NS	NS
Oral contraceptive use	−26.1 (−49.5; −2.68)	**0.030**	NS	NS	NS	NS
Anti‐inflammatory medication	NS	NS	NS	NS	−35.6 (−73.1; 1.90)	0.062
Multivitamin intake	27.7 (4.15; 51.2)	**0.022**	NS	NS	NS	NS
Adj *R* ^2^	0.18		0.051		0.037	
Superoxide dismutase	−1.13 (−2.12; −0.14)	**0.026**	NS	NS	NS	NS
Age	NS	NS	−0.44 (−0.75; −0.13)	**0.005**	NS	NS
Ethnicity	NS	NS	7.91 (2.11; 13.7)	**0.008**	NS	NS
Self‐reported smoking	NS	NS	−8.67 (−17.3; −0.079)	**0.048**	NS	NS
Anti‐inflammatory medication	NS	NS	NS	NS	−35.6 (−73.1; 1.90)	0.062
Antioxidant intake	35.1 (1.40; 68.7)	**0.042**	NS	NS	NS	NS
*Dependent variable Windkessel arterial compliance reactivity (∆%Cwk)*						
Adj *R* ^2^	0.17		NS		NS	
Glutathione peroxidase	0.25 (0.043; 0.47)	**0.019**	NS	NS	NS	NS
Age	−0.36 (−0.71; −0.014)	**0.042**	NS	NS	NS	NS
Multivitamin intake	12.6 (−0.48; 25.6)	0.059	NS	NS	NS	NS
*Dependent variable Stroke volume reactivity (∆%SV)*						
Adj *R* ^2^	NS		0.038		0.036	
Total glutathione	NS	NS	0.008 (0.00; 0.016)	**0.047**	0.027 (−0.002; 0.056)	0.066
Ethnicity	NS	NS	4.61 (1.75; 7.48)	**0.002**	NS	NS

*Note*: All models were adjusted for age, sex, ethnicity, self‐reported smoking status, self‐reported alcohol use, antioxidant and multivitamin intake, anti‐inflammatory medication usage, female hormonal therapy, and oral contraceptive usage. All models were statistically significant (all *p* ≤ 0.026) unless stated otherwise. VIF values were all ≤1.085. Bold values denote statistical significance (*p* < 0.050).

Abbreviations: CI, confidence interval; NS, not significant.

In the mixed‐α/β‐adrenergic responder group, we found u‐NE/Cr to be positively associated with GPx, GR, and GGT (all *p* ≤ 0.036) and inversely associated with NO metabolites (*p* = 0.042). Also, u‐EPI/Cr was positively associated with GPx (*p* = 0.006) in the same responder group. tGSH was positively associated with ∆%SV (*p* = 0.047), and ROS was inversely associated with ∆%CO (*p* = 0.038).

## 4 DISCUSSION

This study describes the interplay of oxidative stress parameters, neuroendocrine markers, and hemodynamic reactivity parameters in predominant α‐ and β‐adrenergic stress reactivity profiles. Similar to previous findings, resting concentrations of neuroendocrine markers and oxidative stress parameters were comparable between predominant α‐ and β‐adrenergic responders after adjusting for multiple confounders. Yet, in fully adjusted regression models, the associations of oxidative stress parameters with neuroendocrine markers and individual hemodynamic reactivity parameters differed across predominant adrenergic reactivity profiles. Specifically in predominant α‐adrenergic responders, oxidative stress may rather be associated with acute stress‐induced hemodynamic reactivity than with neuroendocrine stress‐axis stimulation. In contrast, it is plausible that in predominant β‐adrenergic responders, resting SNS activity might be related to increased glutathione turnover which may reflect an adaptive antioxidant response to increased oxidative stress aimed at preserving cardiac function during stress.

### 4.1 The predominant α‐adrenergic reactivity profile

A predominant α‐adrenergic reactivity profile is characterized by a pattern of excessive peripheral vasoconstriction (increased ∆%TPR) with concomitant decreases in ∆%SV, ∆%CO, and ∆%Cwk (Wentzel et al., 2025) (also reported in Table 1). While a relatively smaller elevation in ∆%HR is observed in predominant α‐adrenergic responders compared to predominant β‐adrenergic responders (Naudé et al., 2026), this hemodynamic stress response pattern imposes a significant burden on the heart which must compensate for an increased afterload (Wentzel et al., 2025). Indeed, predominant α‐adrenergic responders had previously shown a high‐pressure‐related, peripheral vascular risk profile, signified by higher odds for hypertension, cardiac stress, 24‐h ischemic events, and stroke risk probability (Wentzel et al., 2025). Due to the increased cardiac workload, myocardial energy demands increase (Cairns et al., 2025) in which the heart becomes highly reliant on the cardioprotective effects of endogenous antioxidants in response to increased oxidative strain brought upon by acute stress (Xu et al., 2019).

In our current study, resting neuroendocrine markers and oxidative stress parameters did not differ between predominant α‐ and β‐adrenergic responders. In addition, no significant associations were observed between neuroendocrine markers and oxidative stress parameters in the predominant α‐adrenergic responder group. Catecholamines have a dual role in oxidative stress modulation. At normal physiological ranges, catecholamines have shown in experimental studies to display antioxidant properties (Álvarez‐Diduk & Galano, 2015), while during persistent stress exposure, higher levels of catecholamines auto‐oxidize resulting in increased oxidative strain (Sirota, 2020). Indeed, chronic increases in NE concentrations may result in the formation of NE‐derived oxyradicals with superoxide anions as byproducts (Sirota, 2020). These oxidation products have been shown to damage sympathetic nerve terminals in animal studies (Liang et al., 2000) and can ultimately result in cardiac dysfunction (Adameova et al., 2009). Providing protection against oxidative stress, the enzymatic antioxidant SOD scavenges superoxide anions (Fridovich, 1997) and was shown in animal studies to preserve neuronal NE uptake mechanisms and prevent downregulation or uncoupling of myocardial β‐adrenergic receptors caused by chronic NE release (Liang et al., 2000; Mao et al., 2004; Rump & Klaus, 1994; Vatner et al., 1989). Another unique difference to consider is that while neuroendocrine marker levels fluctuate rapidly and transiently (Peaston & Weinkove, 2004), oxidative stress parameters often reflect cumulative oxidative damage (Marrocco et al., 2017). Therefore, despite not including direct markers of oxidative damage in our analyses, it is plausible that the complex and sometimes opposing biochemical roles of catecholamines, together with their differing temporal dynamics during acute stress exposure and how they modulate oxidative stress, may obscure a direct association between circulating catecholamines and systemic oxidative stress parameters in the context of our study. Importantly, our findings reflect acute physiological responses and should not be interpreted as evidence of long‐term of chronic responses to mental stress. Future studies would benefit from longitudinal designs to assess temporal relationships between neuroendocrine markers and oxidative stress parameters across repeated or sustained stress exposures. In addition, ROS measured as serum peroxides in this study may not fully capture tissue‐level redox signaling relevant to cardiovascular pathophysiology which limits mechanistic interpretations at present.

In predominant α‐adrenergic responders, we cautiously propose that oxidative stress may rather be related to inherent characteristics of the predominant α‐adrenergic‐driven hemodynamic pattern including decreased ∆%CO and increased ∆%TPR. Here, it is physiologically viable that enzymatic antioxidants may play a more prominent downstream compensatory role in facilitating these acute stress‐induced hemodynamic alterations observed in this group (see Figure 1a). This is supported by the inverse association observed between SOD and ∆%CO in this group. One possible explanation for this relationship stems from an experimental study indicating that SOD may lower superoxide concentrations, thereby increasing NO bioavailability to promote vasodilation (Vásquez‐Vivar et al., 1998). In the context of our study, it is plausible that such effects could, in part, facilitate sufficient coronary blood flow to sustain lower ∆%CO especially in the setting of increased ∆%TPR and a baseline high‐pressure system. However, given that serum NO metabolite concentrations were comparable between predominant α‐ and β‐adrenergic responders and that no associations were observed with serum NO metabolites in this group, this interpretation cannot be confirmed in this study and warrants further investigation. Although we did not investigate specific isoforms of SOD (Fridovich, 1997), this association could hint at a potential cardioprotective role of SOD during sustained acute stress driven by excessive SNS outflow and predominant α‐adrenergic receptor stimulation (Wentzel et al., 2025).

**FIGURE 1A:**
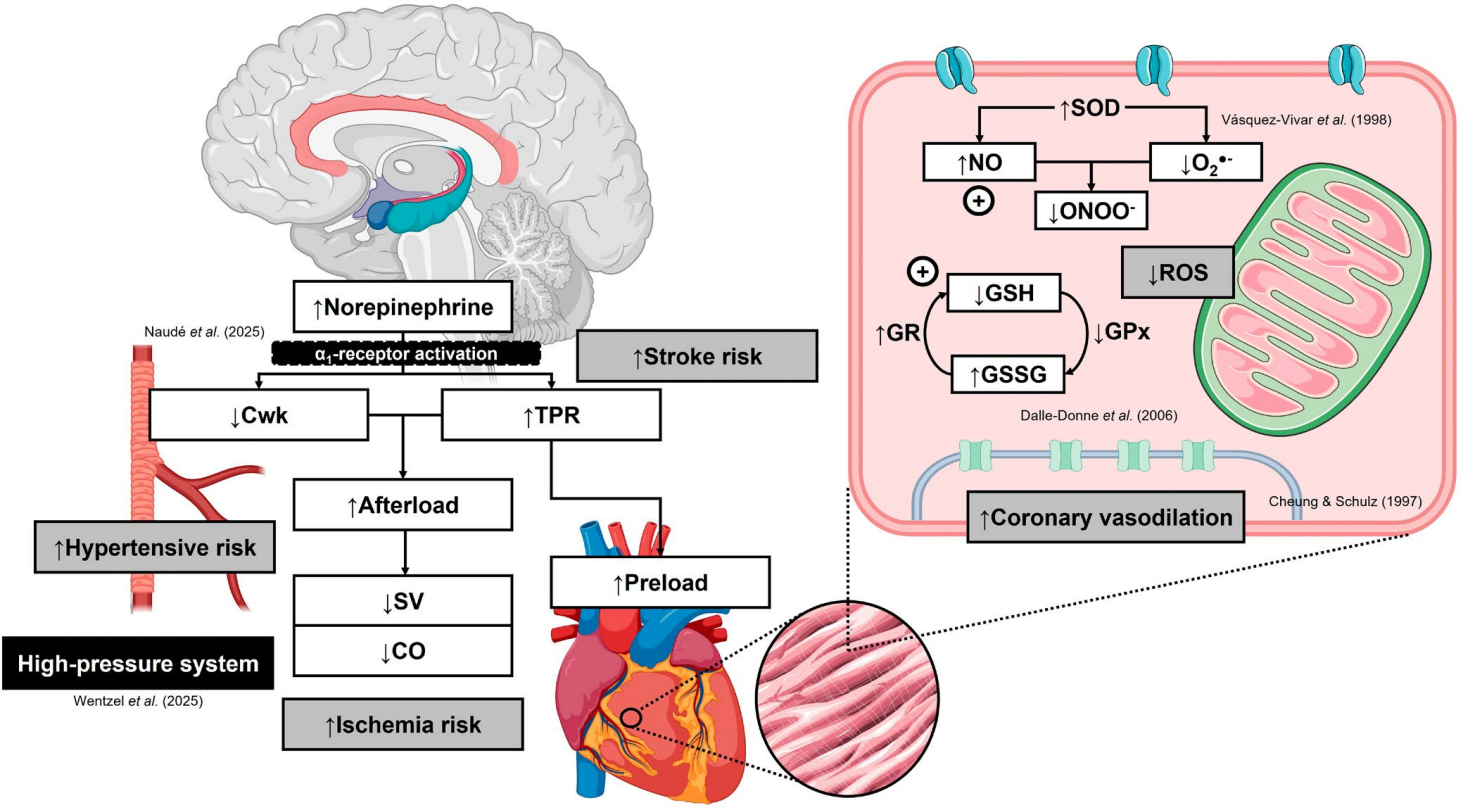
Hypothetical mechanisms indicating the interplay of neuroendocrine markers and oxidative stress parameters with hemodynamic reactivity parameters in predominant α‐adrenergic responders. Ca^2+^, calcium ions; CO, cardiac output; Cwk, Windkessel arterial compliance; GPx, glutathione peroxidase; GR, glutathione reductase; GSSG, oxidized glutathione; O_2_, oxygen; tGSH, total reduced glutathione; ROS, reactive oxygen species; SOD, superoxide dismutase; SV, stroke volume. Figure compiled using elements from BioRender (https://apps.biorender.com) and SMART Servier® Medical Art (Creative Commons Attribution 3.0 Unported License, see https://smart.servier.com).

Additionally, GR showed an inverse association with ∆%CO, while GPx was positively associated with ∆%Cwk. Although no significant group differences in GPx and GR levels were detected, it is possible that in the predominant α‐adrenergic responder group, GR may act as a compensatory marker to regenerate tGSH from its oxidized form (GSSG) (Dalle‐Donne et al., 2006). Enzymatic antioxidants including GPx and GR are known to regulate the reduced‐to‐oxidized glutathione ratio (GSH/GSSG), which is essential for optimal functioning of the glutathione defense system (Zitka et al., 2012). The abovementioned associations observed in this group could possibly relate to previous observations made by Cheung and Schulz (1997) in isolated rat hearts indicating that GSH and GSSG facilitate coronary vasodilation via a nitric oxide‐ and guanylate cyclase‐dependent mechanism (Cheung & Schulz, 1997). In the context of this study, such an observation could possibly aid in preserving cardiac function under conditions of inherently lower Cwk (due to predominant α_1_‐adrenergic activation), especially during bouts of acute stress reactivity. These findings could tentatively suggest that enzymatic antioxidants may potentially play a cardioprotective role in the predominant α‐adrenergic responder group, but further mechanistic studies are required to confirm our hypothesis.

### 4.2 The predominant β‐adrenergic reactivity profile

In predominant β‐adrenergic responders, a positive inotropic effect (increases in ∆%CO and ∆%Cwk; also reported in Table 1) is exerted by NE on cardiomyocytes to enhance blood flow to peripheral organs in order to accommodate increased cardiovascular and metabolic demands imposed by stress (Ginty et al., 2017). We observed a positive association between u‐NE/Cr and GGT which could possibly indicate that resting SNS activity (reflected by preferential β‐adrenergic receptor activation via u‐NE/Cr) may be related to increased glutathione turnover via GGT. Support for this notion comes from in vitro studies indicating that GGT plays a critical role in the extracellular breakdown and recycling of glutathione (Hanigan & Ricketts, 1993; Mitric & Castellano, 2023). As far as we could ascertain, this is the first direct association between greater resting SNS activation (indicated by u‐NE/Cr) and hepatic GGT activity in humans; supporting other studies in human populations which have only found that the liver (which produces GGT) is densely innervated with sympathetic nerve fibers (Falck et al., 1975; Järhult et al., 1977). Interestingly, Loomba et al. (2010) were the first to report an association between plasma GGT secretion and β_2_‐adrenergic receptor (ADRB2) genetic variation in a cohort of White twins (Loomba et al., 2010). Their study suggested that GGT shares significant genetic covariance with several cardiometabolic risk factors (i.e., insulin, HOMA‐IR, triglycerides, and BP) commonly associated with the metabolic syndrome and non‐alcoholic fatty liver disease (NAFLD) which shares one common genetic variation at the ADRB2 locus. Thus, increased adrenergic activity (particularly predominant β_2_‐adrenergic receptor activation) may be involved in key pathophysiological processes associated with the relationship between cardiometabolic risk factors and NAFLD. Despite not observing NAFLD cases in our cohort, the findings of Loomba et al. (2010) could therefore, in part, support the metabolic‐driven cardiometabolic risk profile previously reported cross‐sectionally in predominant β‐adrenergic responders (Wentzel et al., 2025).

Although GGT is often strongly associated with hepatic metabolism and alcohol intake (Whitfield, 2001), we further explored the duality of GGT as a pleiotropic enzyme which is directly associated with glutathione metabolism (Mason et al., 2010). Gamma‐glutamyl transferase is a physiologically viable parameter to include in this study as previous cross‐sectional and longitudinal data in humans have also associated GGT with chronic heart disease and components of the metabolic syndrome, including increased BP and elevated BMI (Grundy, 2007; Lee et al., 2007; Ruttmann et al., 2005). As alluded to earlier, GGT is involved in the breakdown of extracellular tGSH to increase the bioavailability of cysteine, glycine, and glutamic acid (amino acid constituents) for intracellular tGSH synthesis (Mason et al., 2010). Therefore, we carefully suggest that, in response to cases in which transient increases in oxidative stress (ROS possibly derived from sources other than serum peroxides) during acute stress conditions could be observed, tGSH levels may be depleted at a faster rate, potentially resulting in increased GGT expression so as to increase intracellular tGSH levels (see Figure 1b) (Grundy, 2007). As a compensatory effect on cardiac function against the effects of sustained β‐adrenergic activation, we hypothesize that tGSH may promote coronary vasodilation in a similar manner as described earlier by Cheung and Schulz (1997) in isolated rat hearts (Cheung & Schulz, 1997). In turn, this could preserve ∆%CO (and perfusion to peripheral organs (Siddiqui, 2011)) during acute stress experiences which may help prevent possible hyperperfusion injuries to cardiac tissue as previously hypothesized in other work (Fleming, 2010; Harrison et al., 2006; Karbach et al., 2014; Vásquez‐Vivar et al., 1998; Wentzel et al., 2025). Although this hypothesis is further supported by the positive association observed between tGSH and ∆%CO in this responder group, further research is required to confirm. Interestingly, the above‐described hypothetical mechanism involving GGT was previously proposed in a study by Myburgh et al. (2019) in a group who were of Black ethnicity (Myburgh et al., 2019). However, in our study, specifically among predominant β‐adrenergic responders who were primarily of White ethnicity, the same hypothetical mechanism appears to be viable and may therefore be more closely related to the predominant β‐adrenergic reactivity response profile phenotype more so than with ethnicity itself.

**FIGURE 1B:**
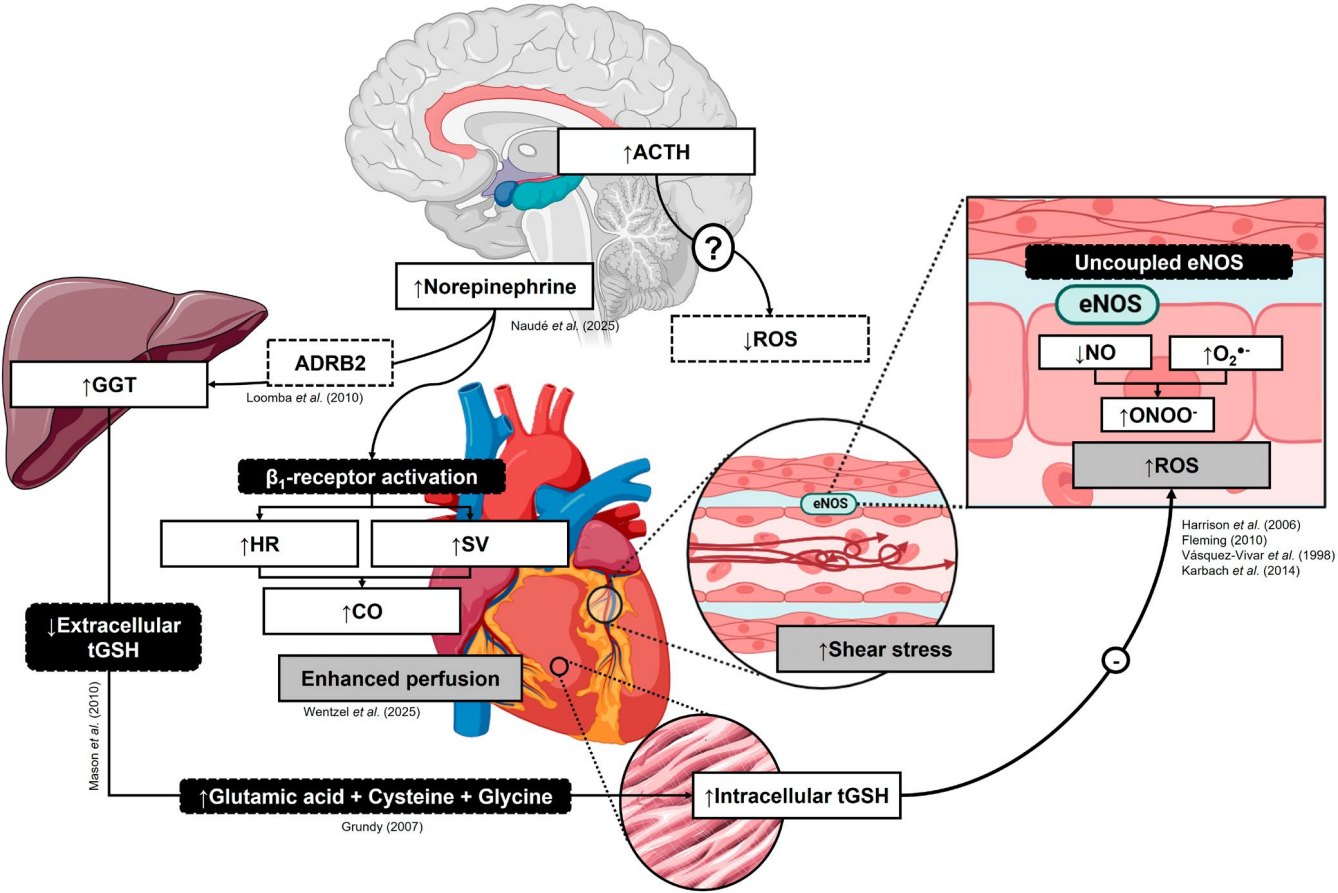
Hypothetical mechanisms indicating the interplay of neuroendocrine markers and oxidative stress parameters with hemodynamic reactivity parameters in predominant β‐adrenergic responders. ACTH, adrenocorticotropic hormone; CO, cardiac output; GGT, gamma‐glutamyl transferase; HR, heart rate; NO, nitric oxide; O_2_^●−^, superoxide; ONOO^−^, peroxynitrate; ROS, reactive oxygen species; SV, stroke volume; tGSH, total glutathione. Figure compiled using elements from BioRender (https://apps.biorender.com) and SMART Servier® Medical Art (Creative Commons Attribution 3.0 Unported License, see https://smart.servier.com).

We further observed an inverse association between ACTH and ROS, but not with cortisol in the predominant β‐adrenergic responder group. The absence of a direct association between cortisol and oxidative stress parameters measured in peripheral blood can possibly be ascribed to the indirect effects of cortisol on oxidative stress modulation. These effects may include changes in glucose metabolism (Ceriello, 2000) or stimulation of anti‐inflammatory mechanisms to decrease cytokine and ROS production (Bjelaković et al., 2007) in order to sufficiently manage the stressful event (Xu et al., 2025). Another explanation could be inactive cortisol in serum, as free (active) cortisol only represents 5%–10% of total plasma/serum cortisol (El‐Farhan et al., 2017; Zuccarella‐Hackl et al., 2024). As resting ACTH, cortisol, and ROS levels were all comparable between predominant α‐ and β‐adrenergic responders, the observed inverse association between ACTH and ROS in our study could be supported by recent findings of Muratoğlu Şahin et al. (2024) who indicated that a direct dose‐dependent relationship between ACTH and oxidative stress modulation exists in humans (Muratoğlu Şahin et al., 2024). In this specific study, the authors demonstrated that ACTH administration, especially at standard doses, rapidly decreases ischemia‐modified albumin levels, which is a biomarker associated with increased ROS production. Despite making use of a different surrogate marker of ROS generation compared to our study, the above‐described findings suggest that there exists a complex interplay between these HPA‐axis markers and oxidative stress in humans which requires further investigation in future studies.

### 4.3 The mixed‐α/β‐adrenergic reactivity profile

The participants who did not fall within the two extreme groups were grouped as mixed‐α/β‐adrenergic responders (Wentzel et al., 2025). Although not the main focus group of this study, these participants were included to describe the most prevalent hemodynamic reactivity response pattern in our population. This responder group had previously shown higher odds for 24‐h hypertension and increased central adiposity only (Wentzel et al., 2025). These individuals also had the highest WC values compared to the two extreme groups and WC itself contributed to the variance of regression models of ACTH and cortisol with ∆%SV (Naudé et al., 2026). These results suggested that the cardiometabolic risk profile related to the mixed‐α/β‐adrenergic responder group is more focused on traditional cardiometabolic risk factors. Our current study additionally reports an inverse association between ROS and ∆%CO in this group which could potentially reflect the well‐described, normal physiological effect of ROS on cardiac functioning. Within the context of previous findings from our group (Naudé et al., 2026), it is plausible that this association may in part be mediated by increased central adiposity. Indeed, obesity‐induced adipose tissue dysfunction may adversely affect adipokine secretion and enhance ROS generation (Zhou et al., 2021). However, future studies may consider mediation analyses with central adiposity measures to further describe the cardiometabolic risk profile associated with this responder group. Based on additional associations observed in this group, we suggest that antioxidants related to the tGSH system may prime the cardiovascular system to respond efficiently during acute stress to ensure sufficient antioxidant defense capacity to regulate ROS production during acute stress. This notion is supported by the observed and expected positive association between tGSH and ∆%SV which may indicate that the normal physiological and cardioprotective role of tGSH (Matuz‐Mares et al., 2021) is also at play (at resting levels) in this responder group. Further research is required to verify our previous hypothesis that the mixed‐α/β‐adrenergic reactivity pattern in part reflects a coordinated hemodynamic response with balanced SNS tone and combined α/β‐adrenergic receptor activation (Naudé et al., 2026).

### 4.4 Strengths, limitations, and future directions

This study should be interpreted in light of its strengths and limitations. Our study sample consisted of schoolteachers from the Dr. Kenneth Kaunda educational district of the North West province and may not fully represent the broader South African population. There may be residual confounding by unquantifiable socio‐environmental or contextual factors due to the significant ethnic disparity among predominant adrenergic responder groups (i.e., predominant α‐adrenergic responders are disproportionately of Black ethnicity and predominant β‐adrenergic responders are primarily White). As a result, we were unable to account for cultural differences or other unmeasured ethnicity‐related influences (e.g., social stress exposure, environmental factors, lifestyle differences) even though ethnicity was included as a confounding factor in all of our analyses. However, participants were all recruited from the same demographic region and from a similar working environment to ensure broadly comparable socioeconomic status, access to healthcare and educational backgrounds. All measurements were also conducted from February to May 2008/2009 in private, temperature‐controlled rooms within the Metabolic Unit in an effort to avoid seasonal changes and to conduct measurements in a well‐controlled clinical environment. Due to the cross‐sectional nature of our study, causal inferences cannot be drawn. Future longitudinal studies should incorporate biomarkers of downstream pathophysiological processes, such as endothelial dysfunction or vascular injury, to determine whether the acute response patterns observed here are associated with longer‐term adaptations or risk trajectories. While findings remained consistent after multiple adjustments, residual confounding cannot be ruled out. As we did not correct for multiple hypothesis testing, these findings should be interpreted as hypothesis‐generating and be confirmed in larger cohorts. Neuroendocrine markers and oxidative stress parameters were measured via standardized clinical procedures in fasting urine and blood samples. Future studies may benefit from assessing neuroendocrine and oxidative stress reactivity in response to acute mental stress within adrenergic reactivity profiles. Researchers are also encouraged to examine catecholamine‐derived oxyradicals and adrenochromes to clarify their role in acute stress‐induced hemodynamic changes across responder groups. As we measured tGSH only, the GSH/GSSG was not assessed. Including this ratio along with markers of oxidative damage (such as TBARS or 8‐oxo‐7,8‐dihydro‐2′‐deoxyguanosine) in future research may offer a more comprehensive evaluation of oxidative adrenergic reactivity profiles. Finally, the study was carefully designed and conducted under controlled conditions, with measures in place to ensure environmental stability.

## 5 CONCLUSION

Our study indicated differential relationships of oxidative stress parameters with neuroendocrine markers and hemodynamic reactivity, depending on the predominant adrenergic reactivity profile phenotype. In predominant α‐adrenergic responders, oxidative stress may be linked to acute stress‐induced hemodynamic reactivity rather than distinct neuroendocrine stress‐axis stimulation. This may suggest that enzymatic antioxidants can possibly facilitate downstream regulation of acute stress‐induced hemodynamic changes linked to high‐pressure‐related peripheral vascular risk. In contrast, in predominant β‐adrenergic responders, resting SNS activity could possibly be related to increased glutathione turnover, which may reflect an adaptive antioxidant response to increased oxidative stress aimed at preserving cardiac function during acute stress experiences.

## AUTHOR CONTRIBUTIONS

**Dewald Naudé:** Conceptualization; data curation; formal analysis; methodology. **Wayne Smith:** Conceptualization; data curation; formal analysis; investigation; methodology; project administration; resources; supervision. **Catharina MC Mels:** Data curation; methodology; project administration; resources; supervision. **Roland von Känel:** Data curation; methodology; project administration; supervision. **Annemarie Wentzel:** Conceptualization; data curation; formal analysis; funding acquisition; investigation; methodology; project administration; resources; software; supervision; validation; visualization.

## FUNDING INFORMATION

This work was supported by North‐West University, the North West Education Department South Africa; Medical Research Council, National Research Foundation South Africa; ROCHE Diagnostic South Africa; Heart and Stroke Foundation South Africa; and the Metabolic Syndrome Institute, France. Any opinions, findings, and conclusions or recommendations expressed in this material are those of the author(s) and therefore funding bodies do not accept any liability in regard thereto.

## CONFLICT OF INTEREST STATEMENT

The authors declare no conflicts of interest.

## DISCLOUSURE

All authors have read and approved the submission of the manuscript; the manuscript has not been published and is not being considered for publication elsewhere, in whole or in part, in any language, except as an abstract. Any opinion, findings, conclusions, or recommendations expressed in this material are those of the authors; therefore, funders do not accept any liability regarding this study. *Use of AI*: No AI tools or software have been used in the compilation of this manuscript aside from the referencing software EndNote.

## Supporting information


**Table S1:** Medical history and medication usage in groups stratified by acute mental stress‐induced adrenergic reactivity profiles (*N* = 362).
**Table S2:** Unadjusted comparisons of neuroendocrine markers and oxidative stress parameters in groups stratified according to acute mental stress‐induced adrenergic reactivity profiles (*N* = 362).
**Table S3:** Spearman rank correlations between reactive oxygen species and various confounders in acute mental stress‐induced adrenergic reactivity profiles (*N* = 362).
**Table S4:** Spearman rank correlations between total glutathione and various confounders in acute mental stress‐induced adrenergic reactivity profiles (*N* = 362).
**Table S5:** Spearman rank correlations between glutathione peroxidase and various confounders in acute mental stress‐induced adrenergic reactivity profiles (*N* = 362).
**Table S6:** Spearman rank correlations between glutathione reductase and various confounders in acute mental stress‐induced adrenergic reactivity profiles (*N* = 362).
**Table S7:** Spearman rank correlations between nitric oxide metabolites and various confounders in acute mental stress‐induced adrenergic reactivity profiles (*N* = 362).
**Table S8:** Spearman rank correlations between superoxide dismutase and various confounders in acute mental stress‐induced adrenergic reactivity profiles (*N* = 362).
**Table S9:** Spearman rank correlations between gamma‐glutamyl transferase and various confounders in acute mental stress‐induced adrenergic reactivity profiles (*N* = 362).

## Data Availability

All inquiries regarding data availability can be made upon reasonable request to the corresponding author, A.W.
