# Supplementary material for: Differential associations of oxidative stress parameters with neuroendocrine markers and hemodynamic reactivity in acute mental stress‐induced adrenergic reactivity profiles: The SABPA study
Source: Physiol Rep. 2026 Jul 21;14(14):e71024. doi: 10.14814/phy2.71024 (PMC13386665; doi:10.14814/phy2.71024)
Supplement: Supplementary file 1 — Table S1: Medical history and medication usage in groups stratified by acute mental stress‐induced adrenergic reactivity profiles (N = 362). Table S2: Unadjusted comparisons of neuroendocrine markers and oxidative stress parameters in groups stratified according to acute mental stress‐induced adrenergic reactivity profiles (N = 362). Table S3: Spearman rank correlations between reactive oxygen species and various confounders in acute mental stress‐induced adrenergic reactivity profiles (N = 362). Table S4: Spearman rank correlations between total glutathione and various confounders in acute mental stress‐induced adrenergic reactivity profiles (N = 362). Table S5: Spearman rank correlations between glutathione peroxidase and various confounders in acute mental stress‐induced adrenergic reactivity profiles (N = 362). Table S6: Spearman rank correlations between glutathione reductase and various confounders in acute mental stress‐induced adrenergic reactivity profiles (N = 362). Table S7: Spearman rank correlations between nitric oxide metabolites and various confounders in acute mental stress‐induced adrenergic reactivity profiles (N = 362). Table S8: Spearman rank correlations between superoxide dismutase and various confounders in acute mental stress‐induced adrenergic reactivity profiles (N = 362). Table S9: Spearman rank correlations between gamma‐glutamyl transferase and various confounders in acute mental stress‐induced adrenergic reactivity profiles (N = 362). [file PHY2-14-e71024-s001.zip › PHYSREP-2026-04-364-T-s02.docx]

| **Table S1:** Medical history and medication usage in groups stratified by acute mental stress-induced adrenergic-haemodynamic reactivity profiles (N=362) | | | | | | | | |  |
| --- | --- | --- | --- | --- | --- | --- | --- | --- | --- |
|  | α-adrenergic  reactivity profile  (n = 47) | | Mixed-α/β-adrenergic  reactivity profile  (n = 247) | | β-adrenergic  reactivity profile  (n = 68) | | *p* | | |
| **Medical history** | | | | | | | | | |
| Stroke, yes, *n (%)* | | 0 (0.0) | | 1 (0.4) | | 0 (0.0) | | 0.79 |  |
| Myocardial infarction, yes, *n (%)* | | 0 (0.0) | | 2 (0.8) | | 1 (1.5) | | 0.69 |  |
| Atrial fibrillation, yes, *n (%)* | | 2 (4.3) | | 9 (3.6) | | 5 (7.4) | | 0.42 |  |
| Kidney disease, yes, *n (%)* | | 0 (0.0) | | 3 (1.2) | | 5 (7.4) | | **0.005** |  |
| Diagnosed diabetes, yes, *n (%)* | | 2 (4.3) | | 9 (3.6) | | 0 (0.0) | | 0.26 |  |
| **Medication** | | | | | | | | | |
| Statins, yes, *n (%)* | | 2 (4.3) | | 7 (2.8) | | 2 (2.9) | | 0.87 |  |
| Acetylcholine agonists, yes, *n (%)* | | 0 (0.0) | | 3 (1.2) | | 0 (0.0) | | 0.49 |  |
| Angiotensin converting enzyme inhibitors, yes, *n (%)* | | 5 (10.6) | | 15 (6.1) | | 2 (2.9) | | 0.24 |  |
| Angiotensin II antagonists, yes, *n (%)* | | 0 (0.0) | | 1 (0.4) | | 0 (0.0) | | 0.79 |  |
| Angiotensin II receptor blockers, yes, *n (%)* | | 0 (0.0) | | 1 (0.4) | | 1 (1.5) | | 0.50 |  |
| Thiazides/Diuretics, yes, *n (%)* | | 5 (10.6) | | 22 (8.9) | | 1 (1.5) | | 0.092 |  |
| Calcium-channel blockers, yes, *n (%)* | | 3 (6.4) | | 10 (4.0) | | 1 (1.5) | | 0.39 |  |
| Beta-adrenergic receptor blockers, yes, *n (%)* | | 1 (2.1) | | 3 (1.2) | | 2 (2.9) | | 0.59 |  |
| Aspirins, yes, *n (%)* | | 2 (4.3) | | 10 (4.0) | | 4 (5.9) | | 0.81 |  |
| Diabetes diet, yes, *n (%)* | | 1 (2.1) | | 12 (4.9) | | 1 (1.5) | | 0.35 |  |
| Oral medication for diabetes, yes, *n (%)* | | 7 (14.9) | | 15 (6.1) | | 3 (4.4) | | 0.061 |  |
| Using insulin for diabetes, yes, *n (%)* | | 3 (6.4) | | 3 (1.2) | | 2 (2.9) | | 0.079 |  |
| Proton pump inhibitors, yes, *n (%)* | | 0 (0.0) | | 0 (0.0) | | 1 (1.5) | | 0.11 |  |
| Anti-microbe drugs, yes, *n (%)* | | 0 (0.0) | | 2 (0.8) | | 1 (1.5) | | 0.69 |  |
| Antihistamine drugs, yes, *n (%)* | | 2 (4.3) | | 7 (2.8) | | 4 (5.9) | | 0.47 |  |
| Insomnia drugs, yes, *n (%)* | | 2 (4.3) | | 1 (0.4) | | 0 (0.0) | | **0.020** |  |
| Anti-coagulant drugs, yes, *n (%)* | | 0 (0.0) | | 2 (0.8) | | 0 (0.0) | | 0.63 |  |
| Antidepressant drugs, yes, *n (%)* | | 1 (2.1) | | 2 (0.8) | | 0 (0.0) | | 0.46 |  |
| Anxiolytic drugs, yes, *n (%)* | | 0 (0.0) | | 2 (0.8) | | 0 (0.0) | | 0.63 |  |
| Anti-epileptic drugs, yes, *n (%)* | | 1 (2.1) | | 1 (0.4) | | 0 (0.0) | | 0.27 |  |
| Sympathetic nervous system blockers, yes, *n (%)* | | 0 (0.0) | | 3 (1.2) | | 0 (0.0) | | 0.49 |  |
| Anti-inflammatory drugs, yes, *n (%)* | | 8 (17.0) | | 12 (4.9) | | 4 (5.9) | | **0.009** |  |
| Cortisone usage, yes, *n (%)* | | 0 (0.0) | | 2 (0.0) | | 0 (0.0) | | 0.63 |  |
| Anti-spasmodic drugs, yes, *n (%)* | | 0 (0.0) | | 2 (0.8) | | 0 (0.0) | | 0.63 |  |
| Analgesic-antipyretic drugs, yes, *n (%)* | | 10 (21.3) | | 23 (9.3) | | 11 (16.2) | | **0.038** |  |
| Paracetamol usage, yes, *n (%)* | | 2 (4.3) | | 10 (4.0) | | 4 (5.9) | | 0.81 |  |
| Thyroxine usage, yes, *n (%)* | | 1 (2.1) | | 1 (0.4) | | 0 (0.0) | | 0.27 |  |
| Contraceptive pill usage, yes, *n (%)* | | 2 (4.3) | | 17 (6.9) | | 3 (4.4) | | 0.64 |  |
| Female hormonal therapy | |  | | | | | | 0.26 |  |
| Oestrogen, yes, *n (%)* | | 2 (4.3) | | 28 (11.3) | | 13 (19.1) | |  |  |
| Progesterone, yes, *n (%)* | | 0 (0.0) | | 3 (1.2) | | 0 (0.0) | |  |  |
| Oestrogen and progesterone, yes, *n (%)* | | 1 (2.1) | | 8 (3.2) | | 2 (2.9) | |  |  |
| Multivitamin intake, yes, *n (%)* | | 2 (4.3) | | 18 (7.3) | | 2 (2.9) | | 0.35 |  |
| Antioxidant intake, yes, *n (%)* | | 1 (2.1) | | 4 (1.6) | | 0 (0.0) | | 0.54 |  |
| Cough syrup (Methylxanthines), yes, *n (%)* | | 0 (0.0) | | 7 (2.8) | | 1 (1.5) | | 0.43 |  |
| Values are expressed as frequency and percentage of participants (*n, %*). Bold values denote p<0.050. All *p*-values were obtained with Chi-square tests. | | | | | | | | |  |
