# Supplementary material for: Differential associations of oxidative stress parameters with neuroendocrine markers and hemodynamic reactivity in acute mental stress‐induced adrenergic reactivity profiles: The SABPA study
Source: Physiol Rep. 2026 Jul 21;14(14):e71024. doi: 10.14814/phy2.71024 (PMC13386665; doi:10.14814/phy2.71024)
Supplement: Supplementary file 1 — Table S1: Medical history and medication usage in groups stratified by acute mental stress‐induced adrenergic reactivity profiles (N = 362). Table S2: Unadjusted comparisons of neuroendocrine markers and oxidative stress parameters in groups stratified according to acute mental stress‐induced adrenergic reactivity profiles (N = 362). Table S3: Spearman rank correlations between reactive oxygen species and various confounders in acute mental stress‐induced adrenergic reactivity profiles (N = 362). Table S4: Spearman rank correlations between total glutathione and various confounders in acute mental stress‐induced adrenergic reactivity profiles (N = 362). Table S5: Spearman rank correlations between glutathione peroxidase and various confounders in acute mental stress‐induced adrenergic reactivity profiles (N = 362). Table S6: Spearman rank correlations between glutathione reductase and various confounders in acute mental stress‐induced adrenergic reactivity profiles (N = 362). Table S7: Spearman rank correlations between nitric oxide metabolites and various confounders in acute mental stress‐induced adrenergic reactivity profiles (N = 362). Table S8: Spearman rank correlations between superoxide dismutase and various confounders in acute mental stress‐induced adrenergic reactivity profiles (N = 362). Table S9: Spearman rank correlations between gamma‐glutamyl transferase and various confounders in acute mental stress‐induced adrenergic reactivity profiles (N = 362). [file PHY2-14-e71024-s001.zip › PHYSREP-2026-04-364-T-s03.docx]

| **Table S2:** Unadjusted comparisons of neuroendocrine markers and oxidative stress parameters in groups stratified according to acute mental stress-induced adrenergic-haemodynamic reactivity profiles (N = 362) | | | | | |
| --- | --- | --- | --- | --- | --- |
|  | α-adrenergic  reactivity profile  (n = 47) | Mixed-α/β-adrenergic  reactivity profile  (n = 247) | β-adrenergic  reactivity profile  (n = 68) | *p* |  |
| **Neuroendocrine markers** | | | | |  |
| Adrenocorticotropic hormone, pg/mL | 17.6 (9.72; 25.9) | 16.5 (11.3; 23.3) | 13.3 (10.0; 20.5) | 0.13 |  |
| Cortisol, nmol/L | 347 (235; 472) | 364 (275; 483)^b^ | 310 (243; 424)^b^ | 0.12 |  |
| u-NE/Cr, nmol/mmol | 23.6 (11.9; 35.3) | 20.8 (11.5; 36.0) | 19.7 (10.3; 33.6) | 0.66 |  |
| u-EPI/Cr, nmol/mmol | 2.62 (1.74; 5.02) | 2.68 (1.60; 4.47) | 2.54 (1.49; 3.81) | 0.74 |  |
| **Oxidative stress markers** | | | | |  |
| Reactive oxygen species, units | 193 (154; 231)^ac^ | 159 (127; 199)^a^ | 146 (118; 180)^c^ | **0.001** |  |
| Total glutathione, μM | 800 (717; 936) | 871 (741; 970) | 828 (714; 1007) | 0.39 |  |
| Glutathione peroxidase, nmol/min/mL | 30.6 (22.4; 38.7)^c^ | 33.4 (28.5; 42.3) | 35.9 (29.8; 40.9)^c^ | 0.055 |  |
| Glutathione reductase, nmol/min/mL | 6.37 (3.82; 10.2)^ac^ | 4.84 (2.80; 7.64)^a^ | 4.33 (2.61; 6.56)^c^ | **0.004** |  |
| Superoxide dismutase, U/mL | 4.35 (3.52; 6.68) | 4.15 (2.88; 5.81) | 4.51 (2.97; 5.94) | 0.40 |  |
| Gamma-glutamyl transferase, U/L | 33.0 (25.0; 71.7)^ac^ | 27.6 (18.0; 50.0)^ab^ | 17.5 (12.3; 30.6)^bc^ | **<0.001** |  |
| Nitric oxide metabolites, μmol/L | 4.88 (1.25; 9.18)^c^ | 2.47 (0.67; 9.23)^b^ | 1.51 (0.53; 4.57)^bc^ | **0.012** |  |
| Data expressed as median (25^th^ and 75^th^ percentiles).  P-values obtained via Kruskal-Wallis tests. Bold values denote statistical significance (p<0.050).  Reactive oxygen species measured as serum peroxides where 1 unit = 1.0 mg/L H_2_O_2_. Nitric oxide metabolites measured as the sum of plasma nitrite and reduced nitrate.  Symbols denote significant differences between ^a^α-adrenergic responders and mixed-adrenergic responders, ^b^β-adrenergic responders and mixed-adrenergic responders and ^c^α-adrenergic responders and β-adrenergic responders were obtained with Dunn-Bonferroni post-hoc tests. | | | | |  |
