# Supplementary material for: Differential associations of oxidative stress parameters with neuroendocrine markers and hemodynamic reactivity in acute mental stress‐induced adrenergic reactivity profiles: The SABPA study
Source: Physiol Rep. 2026 Jul 21;14(14):e71024. doi: 10.14814/phy2.71024 (PMC13386665; doi:10.14814/phy2.71024)
Supplement: Supplementary file 1 — Table S1: Medical history and medication usage in groups stratified by acute mental stress‐induced adrenergic reactivity profiles (N = 362). Table S2: Unadjusted comparisons of neuroendocrine markers and oxidative stress parameters in groups stratified according to acute mental stress‐induced adrenergic reactivity profiles (N = 362). Table S3: Spearman rank correlations between reactive oxygen species and various confounders in acute mental stress‐induced adrenergic reactivity profiles (N = 362). Table S4: Spearman rank correlations between total glutathione and various confounders in acute mental stress‐induced adrenergic reactivity profiles (N = 362). Table S5: Spearman rank correlations between glutathione peroxidase and various confounders in acute mental stress‐induced adrenergic reactivity profiles (N = 362). Table S6: Spearman rank correlations between glutathione reductase and various confounders in acute mental stress‐induced adrenergic reactivity profiles (N = 362). Table S7: Spearman rank correlations between nitric oxide metabolites and various confounders in acute mental stress‐induced adrenergic reactivity profiles (N = 362). Table S8: Spearman rank correlations between superoxide dismutase and various confounders in acute mental stress‐induced adrenergic reactivity profiles (N = 362). Table S9: Spearman rank correlations between gamma‐glutamyl transferase and various confounders in acute mental stress‐induced adrenergic reactivity profiles (N = 362). [file PHY2-14-e71024-s001.zip › Supplementary.docx]

**SUPPLEMENTARY MATERIAL**

| Table S1: Medical history and medication usage in groups stratified by acute mental stress-induced adrenergic reactivity profiles (N=362) | | | | |
| --- | --- | --- | --- | --- |
|  | α-adrenergic  reactivity profile  (n = 47) | Mixed-α/β-adrenergic  reactivity profile  (n = 247) | β-adrenergic  reactivity profile  (n = 68) | *p* |
| **Medical history** | | | | |
| Stroke, yes, *n (%)* | 0 (0.0) | 1 (0.4) | 0 (0.0) | 0.79 |
| Myocardial infarction, yes, *n (%)* | 0 (0.0) | 2 (0.8) | 1 (1.5) | 0.69 |
| Atrial fibrillation, yes, *n (%)* | 2 (4.3) | 9 (3.6) | 5 (7.4) | 0.42 |
| Kidney disease, yes, *n (%)* | 0 (0.0) | 3 (1.2) | 5 (7.4) | **0.005** |
| Diagnosed diabetes, yes, *n (%)* | 2 (4.3) | 9 (3.6) | 0 (0.0) | 0.26 |
| **Medication** | | | | |
| Statins, yes, *n (%)* | 2 (4.3) | 7 (2.8) | 2 (2.9) | 0.87 |
| Acetylcholine agonists, yes, *n (%)* | 0 (0.0) | 3 (1.2) | 0 (0.0) | 0.49 |
| Angiotensin converting enzyme inhibitors, yes, *n (%)* | 5 (10.6) | 15 (6.1) | 2 (2.9) | 0.24 |
| Angiotensin II antagonists, yes, *n (%)* | 0 (0.0) | 1 (0.4) | 0 (0.0) | 0.79 |
| Angiotensin II receptor blockers, yes, *n (%)* | 0 (0.0) | 1 (0.4) | 1 (1.5) | 0.50 |
| Thiazides/Diuretics, yes, *n (%)* | 5 (10.6) | 22 (8.9) | 1 (1.5) | 0.092 |
| Calcium-channel blockers, yes, *n (%)* | 3 (6.4) | 10 (4.0) | 1 (1.5) | 0.39 |
| Beta-adrenergic receptor blockers, yes, *n (%)* | 1 (2.1) | 3 (1.2) | 2 (2.9) | 0.59 |
| Aspirins, yes, *n (%)* | 2 (4.3) | 10 (4.0) | 4 (5.9) | 0.81 |
| Diabetes diet, yes, *n (%)* | 1 (2.1) | 12 (4.9) | 1 (1.5) | 0.35 |
| Oral medication for diabetes, yes, *n (%)* | 7 (14.9) | 15 (6.1) | 3 (4.4) | 0.061 |
| Using insulin for diabetes, yes, *n (%)* | 3 (6.4) | 3 (1.2) | 2 (2.9) | 0.079 |
| Proton pump inhibitors, yes, *n (%)* | 0 (0.0) | 0 (0.0) | 1 (1.5) | 0.11 |
| Anti-microbe drugs, yes, *n (%)* | 0 (0.0) | 2 (0.8) | 1 (1.5) | 0.69 |
| Antihistamine drugs, yes, *n (%)* | 2 (4.3) | 7 (2.8) | 4 (5.9) | 0.47 |
| Insomnia drugs, yes, *n (%)* | 2 (4.3) | 1 (0.4) | 0 (0.0) | **0.020** |
| Anti-coagulant drugs, yes, *n (%)* | 0 (0.0) | 2 (0.8) | 0 (0.0) | 0.63 |
| Antidepressant drugs, yes, *n (%)* | 1 (2.1) | 2 (0.8) | 0 (0.0) | 0.46 |
| Anxiolytic drugs, yes, *n (%)* | 0 (0.0) | 2 (0.8) | 0 (0.0) | 0.63 |
| Anti-epileptic drugs, yes, *n (%)* | 1 (2.1) | 1 (0.4) | 0 (0.0) | 0.27 |
| Sympathetic nervous system blockers, yes, *n (%)* | 0 (0.0) | 3 (1.2) | 0 (0.0) | 0.49 |
| Anti-inflammatory drugs, yes, *n (%)* | 8 (17.0) | 12 (4.9) | 4 (5.9) | **0.009** |
| Cortisone usage, yes, *n (%)* | 0 (0.0) | 2 (0.0) | 0 (0.0) | 0.63 |
| Anti-spasmodic drugs, yes, *n (%)* | 0 (0.0) | 2 (0.8) | 0 (0.0) | 0.63 |
| Analgesic-antipyretic drugs, yes, *n (%)* | 10 (21.3) | 23 (9.3) | 11 (16.2) | **0.038** |
| Paracetamol usage, yes, *n (%)* | 2 (4.3) | 10 (4.0) | 4 (5.9) | 0.81 |
| Thyroxine usage, yes, *n (%)* | 1 (2.1) | 1 (0.4) | 0 (0.0) | 0.27 |
| Contraceptive pill usage, yes, *n (%)* | 2 (4.3) | 17 (6.9) | 3 (4.4) | 0.64 |
| Female hormonal therapy |  | | | 0.26 |
| Estrogen, yes, *n (%)* | 2 (4.3) | 28 (11.3) | 13 (19.1) |  |
| Progesterone, yes, *n (%)* | 0 (0.0) | 3 (1.2) | 0 (0.0) |  |
| Estrogen and progesterone, yes, *n (%)* | 1 (2.1) | 8 (3.2) | 2 (2.9) |  |
| Multivitamin intake, yes, *n (%)* | 2 (4.3) | 18 (7.3) | 2 (2.9) | 0.35 |
| Antioxidant intake, yes, *n (%)* | 1 (2.1) | 4 (1.6) | 0 (0.0) | 0.54 |
| Cough syrup (Methylxanthines), yes, *n (%)* | 0 (0.0) | 7 (2.8) | 1 (1.5) | 0.43 |
| Values are expressed as frequency and percentage of participants (*n, %*). Bold values denote p<0.050. All *p*-values were obtained with Chi-square tests. | | | | |

| Table S2: Unadjusted comparisons of neuroendocrine markers and oxidative stress parameters in groups stratified according to acute mental stress-induced adrenergic reactivity profiles (N = 362) | | | | |  |
| --- | --- | --- | --- | --- | --- |
|  | α-adrenergic  reactivity profile  (n = 47) | Mixed-α/β-adrenergic  reactivity profile  (n = 247) | β-adrenergic  reactivity profile  (n = 68) | *p* | |
| **Neuroendocrine markers** | | | | |  |
| Adrenocorticotropic hormone, pg/mL | 17.6 (9.72; 25.9) | 16.5 (11.3; 23.3) | 13.3 (10.0; 20.5) | 0.13 | |
| Cortisol, nmol/L | 347 (235; 472) | 364 (275; 483)^b^ | 310 (243; 424)^b^ | 0.12 | |
| u-NE/Cr, nmol/mmol | 23.6 (11.9; 35.3) | 20.8 (11.5; 36.0) | 19.7 (10.3; 33.6) | 0.66 | |
| u-EPI/Cr, nmol/mmol | 2.62 (1.74; 5.02) | 2.68 (1.60; 4.47) | 2.54 (1.49; 3.81) | 0.74 | |
| **Oxidative stress parameters** | | | | |  |
| Reactive oxygen species, units | 193 (154; 231)^ac^ | 159 (127; 199)^a^ | 146 (118; 180)^c^ | **0.001** | |
| Total glutathione, μM | 800 (717; 936) | 871 (741; 970) | 828 (714; 1007) | 0.39 | |
| Glutathione peroxidase, nmol/min/mL | 30.6 (22.4; 38.7)^c^ | 33.4 (28.5; 42.3) | 35.9 (29.8; 40.9)^c^ | 0.055 | |
| Glutathione reductase, nmol/min/mL | 6.37 (3.82; 10.2)^ac^ | 4.84 (2.80; 7.64)^a^ | 4.33 (2.61; 6.56)^c^ | **0.004** | |
| Superoxide dismutase, U/mL | 4.35 (3.52; 6.68) | 4.15 (2.88; 5.81) | 4.51 (2.97; 5.94) | 0.40 | |
| Gamma-glutamyl transferase, U/L | 33.0 (25.0; 71.7)^ac^ | 27.6 (18.0; 50.0)^ab^ | 17.5 (12.3; 30.6)^bc^ | **<0.001** | |
| Nitric oxide metabolites, μmol/L | 4.88 (1.25; 9.18)^c^ | 2.47 (0.67; 9.23)^b^ | 1.51 (0.53; 4.57)^bc^ | **0.012** | |
| Data expressed as median (25^th^ and 75^th^ percentiles).  P-values obtained via Kruskal-Wallis tests. Bold values denote statistical significance (p<0.050).  Reactive oxygen species measured as serum peroxides where 1 unit = 1.0 mg/L H_2_O_2_. Nitric oxide metabolites measured as the sum of plasma nitrite and reduced nitrate.  Symbols denote significant differences between ^a^α-adrenergic responders and mixed-adrenergic responders, ^b^β-adrenergic responders and mixed-adrenergic responders and ^c^α-adrenergic responders and β-adrenergic responders were obtained with Dunn-Bonferroni post-hoc tests. | | | | |  |

| Table S3: Spearman rank correlations between reactive oxygen species and various confounders in acute mental stress-induced adrenergic reactivity profiles (N=362) | | | | | | | | |
| --- | --- | --- | --- | --- | --- | --- | --- | --- |
| **Dependent variable: Reactive oxygen** **species** | | | | | | | | |
|  | Total population  (N=362) | | α-adrenergic  reactivity profile (n=47) | | Mixed-α/β-adrenergic  reactivity profile (n=247) | | β-adrenergic  reactivity profile (n=68) | |
|  | r | p | r | p | r | p | r | p |
| Age | 0.088 | 0.095 | 0.069 | 0.65 | 0.061 | 0.34 | 0.040 | 0.75 |
| Sex | **-0.45** | **<0.001** | **-0.42** | **0.004** | **-0.51** | **<0.001** | **-0.33** | **0.006** |
| Ethnicity | **-0.30** | **<0.001** | **-0.43** | **0.002** | **-0.27** | **<0.001** | -0.15 | 0.23 |
| Cotinine | 0.070 | 0.18 | **0.39** | **0.007** | 0.056 | 0.38 | -0.065 | 0.60 |
| Self-reported smoking | 0.005 | 0.92 | -0.035 | 0.81 | 0.012 | 0.85 | 0.039 | 0.75 |
| Self-reported alcohol use | -0.064 | 0.23 | **-0.30** | **0.042** | -0.069 | 0.28 | 0.10 | 0.41 |
| Body mass index | **0.23** | **<0.001** | 0.13 | 0.39 | **0.22** | **0.001** | **0.32** | **0.009** |
| Waist circumference | 0.050 | 0.34 | 0.008 | 0.96 | -0.007 | 0.91 | 0.22 | 0.078 |
| Cardiac output reactivity | **-0.19** | **<0.001** | 0.046 | 0.76 | -0.12 | 0.069 | -0.068 | 0.58 |
| Stroke volume reactivity | **-0.17** | **0.001** | 0.20 | 0.18 | **-0.13** | **0.045** | 0.059 | 0.63 |
| Windkessel arterial compliance reactivity | -0.035 | 0.51 | -0.067 | 0.66 | **0.13** | **0.050** | 0.041 | 0.74 |
| Total peripheral resistance reactivity | **0.19** | **<0.001** | -0.076 | 0.61 | 0.098 | 0.12 | 0.14 | 0.27 |
| Adrenocorticotropic hormone | -0.10 | 0.054 | -0.059 | 0.70 | -0.11 | 0.085 | **-0.24** | **0.049** |
| Cortisol | -0.004 | 0.94 | -0.019 | 0.90 | -0.003 | 0.96 | -0.12 | 0.35 |
| Urinary norepinephrine-to-creatinine ratio | **0.20** | **<0.001** | 0.18 | 0.22 | **0.20** | **0.001** | 0.19 | 0.13 |
| Urinary epinephrine-to-creatinine ratio | 0.063 | 0.23 | 0.048 | 0.75 | 0.041 | 0.52 | 0.11 | 0.39 |
| 24-hour ABPM SBP | 0.081 | 0.12 | -0.098 | 0.51 | 0.026 | 0.68 | 0.096 | 0.43 |
| 24-hour ABPM DBP | 0.055 | 0.30 | -0.11 | 0.48 | 0.010 | 0.87 | 0.092 | 0.46 |
| 24-hour ABPM MAP | 0.068 | 0.19 | -0.13 | 0.37 | 0.019 | 0.77 | 0.085 | 0.49 |
| Glycated hemoglobin | 0.086 | 0.10 | 0.26 | 0.079 | -0.029 | 0.65 | 0.16 | 0.20 |
| Glucose | **-0.14** | **0.008** | -0.14 | 0.35 | **-0.20** | **0.001** | -0.068 | 0.58 |
| Insulin | **0.10** | **0.050** | -0.031 | 0.83 | 0.064 | 0.32 | **0.25** | **0.044** |
| HOMA-IR | 0.057 | 0.28 | -0.009 | 0.95 | -0.011 | 0.87 | **0.25** | **0.044** |
| C-reactive protein | **0.57** | **<0.001** | **0.67** | **<0.001** | **0.55** | **<0.001** | **0.49** | **<0.001** |
| Tumor necrosis factor-alpha | -0.003 | 0.96 | 0.19 | 0.20 | -0.074 | 0.25 | -0.057 | 0.65 |
| Interleukin-6 | **0.32** | **<0.001** | **0.45** | **0.002** | **0.32** | **<0.001** | **0.32** | **0.008** |
| Total cholesterol | -0.056 | 0.29 | 0.008 | 0.96 | -0.11 | 0.075 | 0.15 | 0.21 |
| HDL-cholesterol | **0.11** | **0.030** | 0.15 | 0.30 | 0.11 | 0.074 | 0.16 | 0.19 |
| LDL-cholesterol | -0.079 | 0.13 | 0.025 | 0.87 | **-0.14** | **0.025** | 0.11 | 0.39 |
| Triglycerides | -0.020 | 0.70 | -0.21 | 0.17 | -0.060 | 0.35 | 0.019 | 0.88 |
| Cholesterol-to-HDL ratio | **-0.15** | **0.005** | -0.085 | 0.57 | **-0.21** | **0.001** | -0.041 | 0.74 |
| Estimated glomerular filtration rate | **-0.14** | **0.010** | -0.093 | 0.53 | **-0.16** | **0.011** | -0.058 | 0.64 |
| SNS blockers | -0.026 | 0.62 | - | - | 0.055 | 0.39 | - | - |
| Statins | 0.051 | 0.34 | -0.039 | 0.80 | -0.099 | 0.12 | 0.17 | 0.17 |
| Acetylcholine inhibitors | **0.12** | **0.022** | - | - | 0.060 | 0.35 | - | - |
| ACE inhibitors | 0.061 | 0.25 | 0.12 | 0.41 | **0.16** | **0.012** | -0.21 | 0.081 |
| Angiotensin II antagonist | -0.031 | 0.56 | - | - | 0.073 | 0.25 | - | - |
| Angiotensin receptor blockers | **0.13** | **0.013** | - | - | -0.056 | 0.38 | 0.065 | 0.60 |
| Thiazides/Diuretics | **0.11** | **0.036** | 0.16 | 0.29 | 0.12 | 0.056 | -0.022 | 0.86 |
| Calcium-channel blockers | - | - | 0.038 | 0.80 | **0.13** | **0.038** | -0.009 | 0.94 |
| Alpha-blockers | 0.003 | 0.95 | - | - | - | - | - | - |
| Beta-blockers | 0.094 | 0.074 | 0.19 | 0.21 | -0.040 | 0.53 | 0.075 | 0.54 |
| Aspirins | 0.041 | 0.44 | 0.18 | 0.23 | -0.12 | 0.061 | -0.14 | 0.25 |
| Anti-microbe drugs | -0.025 | 0.63 | - | - | 0.049 | 0.45 | 0.084 | 0.50 |
| Anti-diabetic drugs | 0.037 | 0.49 | 0.25 | 0.092 | -0.087 | 0.18 | - | - |
| Diabetes diet | -0.021 | 0.69 | 0.12 | 0.42 | -0.001 | 0.99 | 0.20 | 0.11 |
| Oral medication for diabetes | -0.025 | 0.63 | 0.022 | 0.88 | -0.11 | 0.091 | 0.19 | 0.13 |
| Using insulin for diabetes | -0.004 | 0.94 | 0.24 | 0.11 | -0.098 | 0.12 | 0.049 | 0.69 |
| Anti-spasmodic drugs | -0.071 | 0.18 | - | - | -0.004 | 0.95 | - | - |
| Proton pump inhibitors | **0.15** | **0.005** | - | - | - | - | -0.17 | 0.18 |
| Anti-inflammatory drugs | 0.046 | 0.38 | 0.28 | 0.053 | 0.089 | 0.17 | 0.13 | 0.28 |
| Anti-coagulant drugs | -0.005 | 0.93 | - | - | 0.056 | 0.38 | - | - |
| Antidepressant drugs | -0.067 | 0.20 | -0.087 | 0.56 | -0.010 | 0.88 | - | - |
| Anxiolytic drugs | -0.019 | 0.72 | - | - | -0.081 | 0.20 | - | - |
| Insomnia drugs | 0.011 | 0.83 | -0.15 | 0.32 | -0.039 | 0.54 | - | - |
| Anti-epileptic drugs | -0.042 | 0.43 | 0.13 | 0.38 | -0.054 | 0.40 | - | - |
| Antihistamine drugs | 0.099 | 0.060 | -0.13 | 0.38 | -0.002 | 0.98 | -0.089 | 0.47 |
| Analgesic-antipyretic drugs | 0.032 | 0.54 | 0.22 | 0.13 | 0.067 | 0.30 | 0.074 | 0.55 |
| Paracetamol usage | -0.046 | 0.38 | 0.26 | 0.073 | 0.043 | 0.50 | -0.083 | 0.50 |
| Thyroxine usage | 0.046 | 0.39 | -0.23 | 0.12 | 0.004 | 0.94 | - | - |
| Cortisone usage | **0.20** | **<0.001** | - | - | 0.055 | 0.39 | - | - |
| Contraceptive pill usage | -0.084 | 0.11 | 0.062 | 0.68 | **0.28** | **<0.001** | -0.060 | 0.63 |
| Female hormonal therapy | - | - | -0.27 | 0.067 | -0.12 | 0.067 | -0.12 | 0.33 |
| Testosterone replacement | -0.003 | 0.96 | - | - | - | - | - | - |
| Multivitamin intake | -0.014 | 0.80 | -0.008 | 0.96 | -0.023 | 0.71 | 0.009 | 0.94 |
| Antioxidant intake | - | - | 0.043 | 0.77 | -0.045 | 0.48 | - | - |
| Anti-retroviral medication | - | - | - | - | - | - | - | - |
| Vertigo treatment | 0.045 | 0.40 | - | - | - | - | - | - |
| Cough syrup | - | - | - | - | 0.032 | 0.62 | 0.15 | 0.23 |
| Correlation coefficients and p-values were obtained with Spearman rank correlation analyses. Bold values denote statistical significance (p<0.050). Abbreviations: ABPM, ambulatory blood pressure monitoring; ACE, angiotensin converting enzyme; DBP, diastolic blood pressure; HDL, high-density lipoprotein; HOMA-IR, homeostatic model assessment for insulin resistance; LDL, low-density lipoprotein; MAP, mean arterial pressure; SBP, systolic blood pressure; SNS, sympathetic nervous system | | | | | | | | |

| Table S4: Spearman rank correlations between total glutathione and various confounders in acute mental stress-induced adrenergic reactivity profiles (N=362) | | | | | | | | |
| --- | --- | --- | --- | --- | --- | --- | --- | --- |
| **Dependent variable: Total glutathione** | | | | | | | | |
|  | Total population  (N=362) | | α-adrenergic  reactivity profile (n=47) | | Mixed-α/β-adrenergic  reactivity profile (n=247) | | β-adrenergic  reactivity profile (n=68) | |
|  | r |  | r | p | r | p | r | p |
| Age | **-0.14** | **0.007** | -0.086 | 0.57 | **-0.21** | **0.001** | 0.11 | 0.37 |
| Sex | **0.23** | **<0.001** | 0.27 | 0.072 | **0.17** | **0.009** | **0.36** | **0.003** |
| Ethnicity | **-0.21** | **<0.001** | -0.10 | 0.49 | **-0.25** | **<0.001** | -0.23 | 0.058 |
| Cotinine | **0.14** | **0.007** | 0.12 | 0.42 | **0.15** | **0.017** | 0.13 | 0.30 |
| Self-reported smoking | **0.16** | **0.002** | 0.20 | 0.17 | **0.16** | **0.012** | 0.13 | 0.28 |
| Self-reported alcohol use | 0.027 | 0.61 | **0.31** | **0.037** | -0.030 | 0.64 | 0.025 | 0.84 |
| Body mass index | 0.018 | 0.73 | 0.008 | 0.96 | 0.055 | 0.39 | -0.098 | 0.43 |
| Waist circumference | 0.054 | 0.31 | 0.001 | 0.99 | 0.095 | 0.14 | -0.044 | 0.72 |
| Cardiac output reactivity | 0.075 | 0.16 | 0.011 | 0.94 | 0.026 | 0.68 | 0.19 | 0.12 |
| Stroke volume reactivity | 0.087 | 0.098 | 0.042 | 0.78 | 0.091 | 0.16 | 0.059 | 0.63 |
| Windkessel arterial compliance reactivity | 0.029 | 0.58 | 0.084 | 0.58 | 0.008 | 0.90 | 0.041 | 0.74 |
| Total peripheral resistance reactivity | -0.054 | 0.30 | -0.073 | 0.63 | -0.005 | 0.94 | 0.14 | 0.27 |
| Adrenocorticotropic hormone | 0.098 | 0.061 | 0.045 | 0.76 | 0.12 | 0.053 | 0.060 | 0.63 |
| Cortisol | **-0.13** | **0.017** | -0.14 | 0.35 | **-0.15** | **0.021** | -0.070 | 0.57 |
| Urinary norepinephrine-to-creatinine ratio | -0.080 | 0.13 | -0.033 | 0.82 | -0.055 | 0.39 | **-0.24** | **0.045** |
| Urinary epinephrine-to-creatinine ratio | 0.053 | 0.32 | 0.099 | 0.51 | 0.035 | 0.59 | 0.085 | 0.49 |
| 24-hour ABPM SBP | **0.11** | **0.035** | 0.13 | 0.39 | 0.12 | 0.069 | 0.22 | 0.068 |
| 24-hour ABPM DBP | **0.16** | **0.003** | 0.22 | 0.14 | **0.15** | **0.023** | 0.21 | 0.089 |
| 24-hour ABPM MAP | **0.14** | **0.010** | 0.22 | 0.14 | **0.13** | **0.040** | 0.21 | 0.081 |
| Glycated hemoglobin | **0.24** | **<0.001** | -0.026 | 0.86 | **0.28** | **<0.001** | **0.35** | **0.004** |
| Glucose | -0.052 | 0.33 | -0.20 | 0.17 | -0.071 | 0.27 | 0.15 | 0.21 |
| Insulin | -0.030 | 0.58 | -0.25 | 0.092 | 0.078 | 0.23 | **-0.28** | **0.021** |
| HOMA-IR | -0.049 | 0.35 | **-0.31** | **0.036** | 0.046 | 0.47 | -0.22 | 0.068 |
| C-reactive protein | -0.058 | 0.27 | -0.005 | 0.98 | -0.029 | 0.65 | -0.11 | 0.35 |
| Tumor necrosis factor-alpha | **0.13** | **0.012** | -0.039 | 0.80 | **0.21** | **0.001** | 0.036 | 0.77 |
| Interleukin-6 | 0.001 | 0.99 | -0.006 | 0.97 | 0.055 | 0.39 | -0.17 | 0.17 |
| Total cholesterol | **-0.14** | **0.006** | -0.22 | 0.14 | **-0.17** | **0.007** | 0.020 | 0.87 |
| HDL-cholesterol | -0.083 | 0.11 | -0.032 | 0.83 | -0.12 | 0.058 | 0.067 | 0.59 |
| LDL-cholesterol | **-0.13** | **0.015** | -0.22 | 0.15 | **-0.15** | **0.016** | 0.011 | 0.93 |
| Triglycerides | -0.031 | 0.56 | -0.078 | 0.60 | -0.041 | 0.52 | 0.062 | 0.62 |
| Cholesterol-to-HDL ratio | -0.014 | 0.79 | -0.11 | 0.48 | -0.003 | 0.97 | -0.020 | 0.87 |
| Estimated glomerular filtration rate | 0.065 | 0.22 | 0.12 | 0.44 | 0.068 | 0.29 | -0.044 | 0.72 |
| SNS blockers | -0.023 | 0.66 | - | - | 0.029 | 0.65 | - | - |
| Statins | 0.054 | 0.31 | -0.12 | 0.44 | 0.022 | 0.73 | -0.089 | 0.47 |
| Acetylcholine inhibitors | 0.060 | 0.26 | - | - | 0.063 | 0.33 | - | - |
| ACE inhibitors | 0.011 | 0.84 | 0.11 | 0.45 | 0.074 | 0.25 | -0.080 | 0.52 |
| Angiotensin II antagonist | -0.079 | 0.13 | - | - | 0.007 | 0.91 | - | - |
| Angiotensin receptor blockers | -0.035 | 0.51 | - | - | -0.059 | 0.36 | -0.15 | 0.21 |
| Thiazides/Diuretics | -0.002 | 0.97 | 0.051 | 0.73 | -0.030 | 0.63 | -0.16 | 0.20 |
| Calcium-channel blockers | - | - | 0.24 | 0.11 | -0.043 | 0.50 | -0.10 | 0.41 |
| Alpha-blockers | 0.064 | 0.23 | - | - | - | - | - | - |
| Beta-blockers | -0.043 | 0.42 | 0.20 | 0.19 | -0.003 | 0.96 | 0.13 | 0.28 |
| Aspirins | 0.065 | 0.22 | -0.13 | 0.38 | -0.072 | 0.26 | -0.18 | 0.15 |
| Anti-microbe drugs | -0.063 | 0.23 | - | - | 0.064 | 0.32 | 0.078 | 0.53 |
| Anti-diabetic drugs | -0.010 | 0.85 | 0.047 | 0.76 | -0.095 | 0.14 | - | - |
| Diabetes diet | -0.020 | 0.70 | 0.25 | 0.090 | -0.027 | 0.67 | -0.17 | 0.18 |
| Oral medication for diabetes | -0.063 | 0.23 | NS | NS | -0.007 | 0.92 | -0.071 | 0.56 |
| Using insulin for diabetes | -0.009 | 0.86 | -0.19 | 0.21 | -0.074 | 0.25 | -0.20 | 0.11 |
| Anti-spasmodic drugs | 0.033 | 0.53 | - | - | -0.023 | 0.71 | - | - |
| Proton pump inhibitors | 0.010 | 0.86 | - | - | - | - | 0.084 | 0.50 |
| Anti-inflammatory drugs | -0.001 | 0.98 | 0.15 | 0.30 | 0.038 | 0.55 | -0.15 | 0.21 |
| Anti-coagulant drugs | -0.021 | 0.69 | - | - | -0.008 | 0.90 | - | - |
| Antidepressant drugs | -0.082 | 0.12 | NS | NS | -0.018 | 0.77 | - | - |
| Anxiolytic drugs | -0.005 | 0.92 | - | - | -0.10 | 0.11 | - | - |
| Insomnia drugs | 0.016 | 0.76 | -0.062 | 0.68 | 0.058 | 0.36 | - | - |
| Anti-epileptic drugs | **-0.13** | **0.013** | 0.12 | 0.42 | -0.022 | 0.73 | - | - |
| Antihistamine drugs | 0.042 | 0.43 | -0.062 | 0.68 | **-0.19** | **0.003** | NS | NS |
| Analgesic-antipyretic drugs | **0.18** | **0.001** | 0.23 | 0.12 | 0.013 | 0.83 | 0.052 | 0.67 |
| Paracetamol usage | 0.017 | 0.75 | 0.20 | 0.17 | **0.13** | **0.050** | **0.33** | **0.006** |
| Thyroxine usage | 0.033 | 0.53 | -0.098 | 0.51 | 0.078 | 0.22 | - | - |
| Cortisone usage | -0.011 | 0.83 | - | - | 0.039 | 0.55 | - | - |
| Contraceptive pill usage | -0.065 | 0.22 | -0.19 | 0.19 | -0.012 | 0.86 | 0.075 | 0.54 |
| Female hormonal therapy | - | - | -0.21 | 0.15 | -0.057 | 0.37 | -0.032 | 0.79 |
| Testosterone replacement | -0.071 | 0.18 | - | - | - | - | - | - |
| Multivitamin intake | -0.059 | 0.27 | -0.016 | 0.92 | -0.11 | 0.082 | 0.067 | 0.59 |
| Antioxidant intake | - | - | 0.043 | 0.77 | -0.081 | 0.21 | - | - |
| Anti-retroviral medication | - | - | - | - | - | - | - | - |
| Vertigo treatment | **0.11** | **0.035** | - | - | - | - | - | - |
| Cough syrup | - | - | - | - | 0.11 | 0.089 | 0.16 | 0.20 |
| Correlation coefficients and p-values were obtained with Spearman rank correlation analyses. Bold values denote statistical significance (p<0.050). Abbreviations: ABPM, ambulatory blood pressure monitoring; ACE, angiotensin converting enzyme; DBP, diastolic blood pressure; HDL, high-density lipoprotein; HOMA-IR, homeostatic model assessment for insulin resistance; LDL, low-density lipoprotein; MAP, mean arterial pressure; SBP, systolic blood pressure; SNS, sympathetic nervous system | | | | | | | | |

| Table S5: Spearman rank correlations between glutathione peroxidase and various confounders in acute mental stress-induced adrenergic reactivity profiles (N=362) | | | | | | | | |
| --- | --- | --- | --- | --- | --- | --- | --- | --- |
| **Dependent variable: Glutathione peroxidase** | | | | | | | | |
|  | Total population  (N=362) | | α-adrenergic  reactivity profile (n=47) | | Mixed-α/β-adrenergic  reactivity profile (n=247) | | β-adrenergic  reactivity profile (n=68) | |
|  | r | p | r | p | r | p | r | p |
| Age | -0.007 | 0.90 | 0.093 | 0.53 | -0.025 | 0.70 | 0.22 | 0.074 |
| Sex | -0.039 | 0.46 | -0.16 | 0.27 | -0.031 | 0.62 | 0.018 | 0.88 |
| Ethnicity | **0.17** | **0.002** | **0.32** | **0.027** | 0.12 | 0.051 | 0.097 | 0.43 |
| Cotinine | 0.005 | 0.93 | 0.11 | 0.48 | -0.006 | 0.93 | -0.032 | 0.79 |
| Self-reported smoking | -0.017 | 0.74 | -0.16 | 0.29 | 0.000 | 1.00 | -0.032 | 0.79 |
| Self-reported alcohol use | 0.003 | 0.95 | -0.14 | 0.35 | 0.034 | 0.60 | -0.042 | 0.74 |
| Body mass index | **-0.14** | **0.007** | 0.078 | 0.60 | **-0.18** | **0.005** | -0.035 | 0.78 |
| Waist circumference | -0.085 | 0.11 | 0.023 | 0.88 | -0.11 | 0.083 | 0.049 | 0.69 |
| Cardiac output reactivity | 0.10 | 0.054 | 0.031 | 0.84 | 0.041 | 0.52 | -0.040 | 0.75 |
| Stroke volume reactivity | 0.12 | 0.12 | 0.074 | 0.62 | 0.066 | 0.30 | 0.086 | 0.49 |
| Windkessel arterial compliance reactivity | 0.089 | 0.090 | **0.33** | **0.024** | 0.005 | 0.93 | -0.058 | 0.64 |
| Total peripheral resistance reactivity | -0.099 | 0.059 | -0.098 | 0.51 | -0.047 | 0.47 | -0.13 | 0.28 |
| Adrenocorticotropic hormone | -0.096 | 0.068 | -0.25 | 0.093 | -0.090 | 0.16 | 0.075 | 0.55 |
| Cortisol | -0.076 | 0.15 | **-0.33** | **0.025** | -0.077 | 0.23 | 0.11 | 0.39 |
| Urinary norepinephrine-to-creatinine ratio | **0.19** | **<0.001** | **0.29** | **0.050** | **0.18** | **0.005** | 0.18 | 0.15 |
| Urinary epinephrine-to-creatinine ratio | **0.18** | **0.001** | 0.061 | 0.69 | **0.20** | **0.002** | 0.17 | 0.18 |
| 24-hour ABPM SBP | **-0.15** | **0.004** | **-0.52** | **<0.001** | -0.078 | 0.22 | 0.058 | 0.64 |
| 24-hour ABPM DBP | **-0.11** | **0.044** | **-0.43** | **0.003** | -0.057 | 0.37 | 0.085 | 0.49 |
| 24-hour ABPM MAP | **-0.13** | **0.013** | **-0.51** | **<0.001** | -0.071 | 0.27 | 0.094 | 0.45 |
| Glycated hemoglobin | **-0.10** | **0.050** | -0.12 | 0.44 | -0.054 | 0.40 | -0.21 | 0.093 |
| Glucose | -0.047 | 0.38 | -0.13 | 0.37 | -0.004 | 0.95 | -0.054 | 0.66 |
| Insulin | **-0.12** | **0.019** | -0.067 | 0.65 | -0.12 | 0.054 | -0.13 | 0.30 |
| HOMA-IR | **-0.11** | **0.039** | -0.035 | 0.81 | -0.098 | 0.13 | -0.17 | 0.17 |
| C-reactive protein | **-0.11** | **0.043** | 0.13 | 0.40 | -0.12 | 0.061 | -0.048 | 0.70 |
| Tumor necrosis factor-alpha | 0.093 | 0.079 | -0.005 | 0.97 | 0.12 | 0.061 | 0.20 | 0.11 |
| Interleukin-6 | -0.046 | 0.38 | 0.047 | 0.76 | -0.067 | 0.29 | 0.042 | 0.74 |
| Total cholesterol | 0.025 | 0.64 | 0.12 | 0.43 | 0.050 | 0.43 | -0.15 | 0.23 |
| HDL-cholesterol | 0.040 | 0.45 | 0.058 | 0.70 | 0.049 | 0.45 | -0.030 | 0.81 |
| LDL-cholesterol | 0.000 | 0.99 | 0.13 | 0.38 | 0.015 | 0.82 | -0.16 | 0.21 |
| Triglycerides | 0.019 | 0.73 | -0.13 | 0.40 | 0.079 | 0.22 | -0.001 | 0.99 |
| Cholesterol-to-HDL ratio | -0.016 | 0.76 | -0.012 | 0.94 | -0.007 | 0.92 | -0.042 | 0.73 |
| Estimated glomerular filtration rate | 0.029 | 0.58 | 0.023 | 0.88 | 0.038 | 0.55 | -0.024 | 0.85 |
| SNS blockers | 0.026 | 0.62 | - | - | 0.005 | 0.94 | - | - |
| Statins | 0.047 | 0.37 | 0.11 | 0.48 | -0.018 | 0.77 | 0.16 | 0.21 |
| Acetylcholine inhibitors | 0.076 | 0.15 | - | - | 0.053 | 0.41 | - | - |
| ACE inhibitors | 0.009 | 0.86 | 0.12 | 0.43 | 0.11 | 0.10 | -0.031 | 0.80 |
| Angiotensin II antagonist | -0.021 | 0.69 | - | - | 0.013 | 0.83 | - | - |
| Angiotensin receptor blockers | 0.024 | 0.65 | - | - | -0.065 | 0.31 | 0.037 | 0.76 |
| Thiazides/Diuretics | 0.017 | 0.74 | 0.17 | 0.25 | 0.018 | 0.78 | -0.11 | 0.39 |
| Calcium-channel blockers | - | - | 0.25 | 0.090 | -0.007 | 0.91 | -0.11 | 0.39 |
| Alpha-blockers | -0.081 | 0.13 | - | - | - | - | - | - |
| Beta-blockers | -0.031 | 0.55 | -0.16 | 0.27 | **-0.13** | **0.050** | 0.062 | 0.62 |
| Aspirins | 0.008 | 0.88 | 0.062 | 0.68 | 0.031 | 0.62 | -0.083 | 0.50 |
| Anti-microbe drugs | 0.054 | 0.31 | - | - | -0.042 | 0.51 | 0.15 | 0.23 |
| Anti-diabetic drugs | -0.057 | 0.28 | 0.25 | 0.087 | 0.028 | 0.67 | - | - |
| Diabetes diet | 0.073 | 0.17 | 0.13 | 0.38 | -0.095 | 0.14 | NS | NS |
| Oral medication for diabetes | 0.054 | 0.31 | 0.24 | 0.10 | 0.056 | 0.38 | 0.015 | 0.91 |
| Using insulin for diabetes | -0.081 | 0.12 | 0.061 | 0.68 | 0.050 | 0.43 | -0.038 | 0.76 |
| Anti-spasmodic drugs | 0.041 | 0.44 | - | - | -0.098 | 0.13 | - | - |
| Proton pump inhibitors | 0.036 | 0.50 | - | - | - | - | 0.087 | 0.48 |
| Anti-inflammatory drugs | -0.052 | 0.33 | 0.24 | 0.10 | NS | NS | 0.097 | 0.43 |
| Anti-coagulant drugs | 0.032 | 0.55 | - | - | -0.060 | 0.35 | - | - |
| Antidepressant drugs | 0.005 | 0.93 | NS | NS | 0.061 | 0.34 | - | - |
| Anxiolytic drugs | -0.062 | 0.24 | - | - | 0.006 | 0.93 | - | - |
| Insomnia drugs | 0.038 | 0.47 | -0.023 | 0.88 | -0.074 | 0.25 | - | - |
| Anti-epileptic drugs | -0.006 | 0.91 | 0.20 | 0.19 | -0.005 | 0.93 | - | - |
| Antihistamine drugs | 0.017 | 0.74 | 0.074 | 0.62 | -0.009 | 0.89 | -0.064 | 0.61 |
| Analgesic-antipyretic drugs | **0.10** | **0.049** | -0.10 | 0.49 | 0.029 | 0.65 | 0.18 | 0.15 |
| Paracetamol usage | 0.048 | 0.36 | 0.062 | 0.68 | **0.14** | **0.032** | 0.025 | 0.84 |
| Thyroxine usage | **0.12** | **0.024** | 0.12 | 0.42 | 0.041 | 0.52 | - | - |
| Cortisone usage | -0.079 | 0.13 | - | - | **0.14** | **0.028** | - | - |
| Contraceptive pill usage | 0.040 | 0.45 | -0.082 | 0.59 | -0.086 | 0.18 | -0.060 | 0.63 |
| Female hormonal therapy | - | - | -0.088 | 0.56 | 0.021 | 0.75 | 0.22 | 0.077 |
| Testosterone replacement | 0.048 | 0.36 | - | - | - | - | - | - |
| Multivitamin intake | 0.001 | 0.98 | 0.031 | 0.84 | 0.091 | 0.15 | -0.20 | 0.095 |
| Antioxidant intake | - | - | - | - | -0.029 | 0.65 | - | - |
| Anti-retroviral medication | - | - | - | - | - | - | - | - |
| Vertigo treatment | -0.037 | 0.49 | - | - | - | - | - | - |
| Cough syrup | - | - | - | - | -0.066 | 0.30 | 0.078 | 0.53 |
| Correlation coefficients and p-values were obtained with Spearman rank correlation analyses. Bold values denote statistical significance (p<0.050). Abbreviations: ABPM, ambulatory blood pressure monitoring; ACE, angiotensin converting enzyme; DBP, diastolic blood pressure; HDL, high-density lipoprotein; HOMA-IR, homeostatic model assessment for insulin resistance; LDL, low-density lipoprotein; MAP, mean arterial pressure; SBP, systolic blood pressure; SNS, sympathetic nervous system | | | | | | | | |

| Table S6: Spearman rank correlations between glutathione reductase and various confounders in acute mental stress-induced adrenergic reactivity profiles (N=362) | | | | | | | | |
| --- | --- | --- | --- | --- | --- | --- | --- | --- |
| **Dependent variable: Glutathione reductase** | | | | | | | | |
|  | Total population  (N=362) | | α-adrenergic  reactivity profile (n=47) | | Mixed-α/β-adrenergic  reactivity profile (n=247) | | β-adrenergic  reactivity profile (n=68) | |
|  | r | p | r | p | r | p | r | p |
| Age | 0.063 | 0.23 | -0.010 | 0.95 | -0.012 | 0.86 | 0.18 | 0.15 |
| Sex | -0.040 | 0.45 | **0.29** | **0.046** | **-0.14** | **0.032** | 0.042 | 0.74 |
| Ethnicity | **-0.56** | **<0.001** | **-0.62** | **<0.001** | **-0.54** | **<0.001** | -0.49 | **<0.001** |
| Cotinine | 0.069 | 0.19 | **0.31** | **0.036** | -0.001 | 0.98 | 0.11 | 0.37 |
| Self-reported smoking | 0.009 | 0.87 | **0.32** | **0.027** | -0.077 | 0.23 | 0.097 | 0.43 |
| Self-reported alcohol use | -0.090 | 0.088 | -0.28 | 0.054 | -0.003 | 0.96 | **-0.28** | **0.020** |
| Body mass index | **0.13** | **0.012** | **0.30** | **0.042** | 0.052 | 0.42 | 0.23 | 0.055 |
| Waist circumference | 0.033 | 0.54 | **0.37** | **0.011** | -0.082 | 0.20 | 0.15 | 0.24 |
| Cardiac output reactivity | **-0.17** | **<0.001** | -0.24 | 0.098 | -0.037 | 0.57 | -0.19 | 0.13 |
| Stroke volume reactivity | **-0.17** | **<0.001** | -0.049 | 0.74 | -0.11 | 0.077 | 0.034 | 0.78 |
| Windkessel arterial compliance reactivity | -0.085 | 0.11 | -0.17 | 0.26 | 0.031 | 0.62 | 0.092 | 0.46 |
| Total peripheral resistance reactivity | **0.17** | **0.001** | 0.14 | 0.32 | 0.048 | 0.46 | 0.17 | 0.17 |
| Adrenocorticotropic hormone | 0.085 | 0.11 | **0.34** | **0.020** | 0.002 | 0.97 | 0.13 | 0.29 |
| Cortisol | -0.073 | 0.17 | -0.044 | 0.77 | -0.10 | 0.11 | -0.11 | 0.39 |
| Urinary norepinephrine-to-creatinine ratio | 0.058 | 0.27 | -0.038 | 0.80 | 0.099 | 0.12 | -0.084 | 0.50 |
| Urinary epinephrine-to-creatinine ratio | **0.13** | **0.014** | 0.21 | 0.16 | **0.14** | **0.029** | -0.019 | 0.88 |
| 24-hour ABPM SBP | **0.17** | **0.001** | 0.25 | 0.096 | 0.096 | 0.13 | 0.11 | 0.38 |
| 24-hour ABPM DBP | **0.17** | **0.002** | 0.24 | 0.099 | **0.13** | **0.047** | 0.047 | 0.71 |
| 24-hour ABPM MAP | **0.18** | **0.001** | 0.26 | 0.076 | 0.11 | 0.073 | 0.089 | 0.47 |
| Glycated hemoglobin | **0.16** | **0.003** | **0.34** | **0.019** | 0.043 | 0.50 | 0.19 | 0.12 |
| Glucose | **-0.10** | **0.048** | 0.17 | 0.27 | **-0.19** | **0.003** | -0.22 | 0.075 |
| Insulin | **0.15** | **0.006** | **0.45** | **0.002** | 0.032 | 0.62 | 0.23 | 0.057 |
| HOMA-IR | **0.13** | **0.016** | **0.43** | **0.002** | 0.006 | 0.92 | 0.18 | 0.15 |
| C-reactive protein | **0.30** | **<0.001** | **0.30** | **0.041** | **0.28** | **<0.001** | **0.28** | **0.021** |
| Tumor necrosis factor-alpha | **0.18** | **<0.001** | **0.38** | **0.009** | 0.11 | 0.086 | 0.24 | 0.051 |
| Interleukin-6 | **0.25** | **<0.001** | 0.22 | 0.14 | **0.21** | **0.001** | **0.31** | **0.010** |
| Total cholesterol | **-0.13** | **0.012** | -0.23 | 0.12 | **-0.14** | **0.033** | 0.044 | 0.72 |
| HDL-cholesterol | -0.027 | 0.61 | **-0.31** | **0.036** | 0.085 | 0.18 | -0.19 | 0.13 |
| LDL-cholesterol | **-0.16** | **0.002** | -0.24 | 0.098 | **-0.19** | **0.003** | 0.050 | 0.68 |
| Triglycerides | **0.15** | **0.004** | **0.32** | **0.029** | 0.049 | 0.45 | **0.30** | **0.014** |
| Cholesterol-to-HDL ratio | -0.080 | 0.13 | 0.073 | 0.63 | **-0.18** | **0.004** | 0.16 | 0.19 |
| Estimated glomerular filtration rate | -0.053 | 0.32 | -0.068 | 0.65 | -0.041 | 0.52 | -0.004 | 0.97 |
| SNS blockers | -0.051 | 0.34 | - | - | 0.091 | 0.16 | - | - |
| Statins | 0.062 | 0.24 | -0.019 | 0.90 | -0.084 | 0.19 | 0.027 | 0.83 |
| Acetylcholine inhibitors | **0.12** | **0.019** | - | - | 0.075 | 0.24 | - | - |
| ACE inhibitors | -0.066 | 0.21 | 0.13 | 0.38 | 0.12 | 0.054 | -0.024 | 0.84 |
| Angiotensin II antagonist | -0.059 | 0.27 | - | - | -0.077 | 0.23 | - | - |
| Angiotensin receptor blockers | 0.097 | 0.066 | - | - | -0.11 | 0.095 | 0.056 | 0.65 |
| Thiazides/Diuretics | **0.13** | **0.017** | 0.14 | 0.34 | 0.065 | 0.31 | 0.17 | 0.17 |
| Calcium-channel blockers | - | - | 0.12 | 0.41 | **0.13** | **0.044** | 0.009 | 0.94 |
| Alpha-blockers | 0.049 | 0.35 | - | - | - | - | - | - |
| Beta-blockers | 0.23 | **<0.001** | 0.054 | 0.72 | 0.083 | 0.19 | -0.053 | 0.67 |
| Aspirins | 0.039 | 0.47 | 0.051 | 0.74 | -0.032 | 0.62 | 0.13 | 0.31 |
| Anti-microbe drugs | **0.11** | **0.030** | - | - | 0.10 | 0.11 | -0.12 | 0.34 |
| Anti-diabetic drugs | 0.075 | 0.15 | 0.14 | 0.36 | 0.099 | 0.12 | - | - |
| Diabetes diet | **0.12** | **0.029** | 0.076 | 0.61 | 0.094 | 0.14 | -0.093 | 0.45 |
| Oral medication for diabetes | **0.11** | **0.030** | 0.007 | 0.97 | 0.10 | 0.10 | 0.10 | 0.42 |
| Using insulin for diabetes | 0.094 | 0.074 | 0.045 | 0.76 | 0.088 | 0.17 | -0.13 | 0.28 |
| Anti-spasmodic drugs | 0.025 | 0.64 | - | - | 0.11 | 0.078 | - | - |
| Proton pump inhibitors | 0.060 | 0.25 | - | - | - | - | 0.10 | 0.42 |
| Anti-inflammatory drugs | 0.087 | 0.098 | -0.15 | 0.33 | 0.060 | 0.34 | 0.19 | 0.12 |
| Anti-coagulant drugs | -0.029 | 0.59 | - | - | 0.10 | 0.10 | - | - |
| Antidepressant drugs | NS | NS | -0.19 | 0.20 | -0.001 | 0.99 | - | - |
| Anxiolytic drugs | **-0.12** | **0.024** | - | - | -0.001 | 0.99 | - | - |
| Insomnia drugs | -0.015 | 0.77 | **-0.32** | **0.031** | -0.082 | 0.20 | - | - |
| Anti-epileptic drugs | -0.050 | 0.35 | 0.016 | 0.91 | -0.057 | 0.37 | - | - |
| Antihistamine drugs | -0.042 | 0.43 | -0.054 | 0.72 | -0.028 | 0.67 | -0.12 | 0.31 |
| Analgesic-antipyretic drugs | -0.054 | 0.30 | -0.19 | 0.20 | -0.030 | 0.64 | 0.004 | 0.97 |
| Paracetamol usage | 0.011 | 0.83 | -0.22 | 0.14 | -0.044 | 0.49 | 0.049 | 0.67 |
| Thyroxine usage | -0.015 | 0.78 | 0.043 | 0.77 | -0.032 | 0.62 | - | - |
| Cortisone usage | -0.006 | 0.92 | - | - | -0.017 | 0.79 | - | - |
| Contraceptive pill usage | -0.050 | 0.34 | -0.031 | 0.84 | 0.029 | 0.65 | -0.17 | 0.16 |
| Female hormonal therapy | - | - | 0.11 | 0.45 | -0.11 | 0.087 | -0.035 | 0.78 |
| Testosterone replacement | -0.100 | 0.056 | - | - | - | - | - | - |
| Multivitamin intake | **-0.11** | **0.046** | 0.070 | 0.64 | -0.11 | 0.086 | **-0.26** | **0.036** |
| Antioxidant intake | - | - | -0.087 | 0.56 | **-0.14** | **0.033** | - | - |
| Anti-retroviral medication | - | - | - | - | - | - | - | - |
| Vertigo treatment | **0.14** | **0.009** | - | - | - | - | - | - |
| Cough syrup | - | - | - | - | **0.18** | **0.006** | 0.031 | 0.80 |
| Correlation coefficients and p-values were obtained with Spearman rank correlation analyses. Bold values denote statistical significance (p<0.050). Abbreviations: ABPM, ambulatory blood pressure monitoring; ACE, angiotensin converting enzyme; DBP, diastolic blood pressure; HDL, high-density lipoprotein; HOMA-IR, homeostatic model assessment for insulin resistance; LDL, low-density lipoprotein; MAP, mean arterial pressure; SBP, systolic blood pressure; SNS, sympathetic nervous system | | | | | | | | |

| Table S7: Spearman rank correlations between nitric oxide metabolites and various confounders in acute mental stress-induced adrenergic reactivity profiles (N=362) | | | | | | | | |
| --- | --- | --- | --- | --- | --- | --- | --- | --- |
| **Dependent variable: Nitric oxide metabolites** | | | | | | | | |
|  | Total population  (N=362) | | α-adrenergic  reactivity profile (n=47) | | Mixed-α/β-adrenergic  reactivity profile (n=247) | | β-adrenergic  reactivity profile (n=68) | |
|  | r | p | r | p | r | p | r | p |
| Age | -0.039 | 0.46 | 0.058 | 0.70 | -0.10 | 0.11 | -0.027 | 0.82 |
| Sex | -0.061 | 0.25 | 0.095 | 0.53 | **-0.13** | **0.040** | 0.078 | 0.53 |
| Ethnicity | **-0.60** | **<0.001** | **-0.55** | **<0.001** | **-0.61** | **<0.001** | **-0.39** | **0.001** |
| Cotinine | **0.15** | **0.006** | 0.27 | 0.063 | **0.13** | **0.044** | 0.078 | 0.53 |
| Self-reported smoking | -0.002 | 0.97 | 0.17 | 0.25 | -0.040 | 0.53 | 0.060 | 0.63 |
| Self-reported alcohol use | **-0.18** | **0.001** | -0.096 | 0.52 | **-0.22** | **0.001** | -0.057 | 0.64 |
| Body mass index | **0.18** | **0.001** | 0.046 | 0.76 | **0.17** | **0.009** | 0.17 | 0.16 |
| Waist circumference | 0.034 | 0.51 | -0.047 | 0.75 | 0.004 | 0.94 | 0.076 | 0.54 |
| Cardiac output reactivity | **-0.19** | **<0.001** | 0.052 | 0.73 | **-0.13** | **0.038** | -0.072 | 0.56 |
| Stroke volume reactivity | **-0.13** | **0.014** | **0.32** | **0.027** | -0.067 | 0.29 | -0.080 | 0.52 |
| Windkessel arterial compliance reactivity | -0.088 | 0.094 | 0.036 | 0.81 | 0.011 | 0.86 | 0.002 | 0.98 |
| Total peripheral resistance reactivity | **0.22** | **<0.001** | -0.10 | 0.49 | **0.16** | **0.012** | 0.15 | 0.23 |
| Adrenocorticotropic hormone | **0.12** | **0.029** | **0.31** | **0.034** | 0.047 | 0.46 | 0.19 | 0.13 |
| Cortisol | -0.098 | 0.063 | -0.078 | 0.60 | **-0.14** | **0.028** | -0.045 | 0.71 |
| Urinary norepinephrine-to-creatinine ratio | **-0.16** | **0.002** | -0.14 | 0.35 | **-0.15** | **0.022** | **-0.26** | **0.035** |
| Urinary epinephrine-to-creatinine ratio | 0.079 | 0.13 | 0.25 | 0.097 | 0.046 | 0.47 | 0.070 | 0.57 |
| 24-hour ABPM SBP | **0.11** | **0.038** | 0.14 | 0.35 | 0.035 | 0.59 | 0.15 | 0.21 |
| 24-hour ABPM DBP | **0.15** | **0.005** | 0.17 | 0.25 | 0.085 | 0.18 | 0.19 | 0.12 |
| 24-hour ABPM MAP | **0.14** | **0.007** | 0.20 | 0.18 | 0.065 | 0.31 | 0.20 | 0.11 |
| Glycated hemoglobin | **0.22** | **<0.001** | 0.28 | 0.054 | **0.17** | **0.006** | 0.17 | 0.16 |
| Glucose | **-0.13** | **0.012** | -0.018 | 0.90 | **-0.17** | **0.007** | **-0.26** | **0.030** |
| Insulin | 0.067 | 0.20 | 0.006 | 0.97 | 0.032 | 0.61 | 0.066 | 0.59 |
| HOMA-IR | 0.045 | 0.39 | 0.018 | 0.91 | 0.001 | 0.99 | 0.036 | 0.77 |
| C-reactive protein | **0.28** | **<0.001** | **0.32** | **0.030** | **0.22** | **0.001** | **0.37** | **0.002** |
| Tumor necrosis factor-alpha | 0.10 | 0.056 | 0.20 | 0.19 | 0.074 | 0.25 | 0.049 | 0.69 |
| Interleukin-6 | **0.25** | **<0.001** | **0.32** | **0.031** | **0.22** | **0.001** | **0.32** | **0.007** |
| Total cholesterol | **-0.24** | **<0.001** | -0.21 | 0.15 | **-0.26** | **<0.001** | -0.093 | 0.45 |
| HDL-cholesterol | -0.074 | 0.16 | -0.069 | 0.65 | -0.038 | 0.55 | -0.19 | 0.13 |
| LDL-cholesterol | **-0.25** | **<0.001** | -0.23 | 0.12 | **-0.28** | **<0.001** | -0.091 | 0.46 |
| Triglycerides | 0.029 | 0.58 | 0.062 | 0.68 | -0.024 | 0.71 | 0.064 | 0.60 |
| Cholesterol-to-HDL ratio | **-0.11** | **0.041** | -0.053 | 0.72 | **-0.15** | **0.016** | 0.002 | 0.99 |
| Estimated glomerular filtration rate | -0.053 | 0.32 | 0.084 | 0.58 | -0.069 | 0.28 | -0.075 | 0.55 |
| SNS blockers | -0.058 | 0.27 | - | - | -0.030 | 0.64 | - | - |
| Statins | 0.089 | 0.092 | -0.18 | 0.23 | -0.016 | 0.80 | -0.16 | 0.19 |
| Acetylcholine inhibitors | **0.11** | **0.032** | - | - | 0.10 | 0.20 | - | - |
| ACE inhibitors | NS | NS | -0.056 | 0.71 | **0.15** | **0.022** | 0.033 | 0.79 |
| Angiotensin II antagonist | -0.057 | 0.28 | - | - | -0.005 | 0.94 | - | - |
| Angiotensin receptor blockers | **0.15** | **0.005** | - | - | 0.008 | 0.90 | -0.21 | 0.088 |
| Thiazides/Diuretics | 0.066 | 0.21 | 0.25 | 0.088 | 0.12 | 0.072 | 0.16 | 0.20 |
| Calcium-channel blockers | - | - | -0.096 | 0.52 | 0.067 | 0.29 | 0.15 | 0.21 |
| Alpha-blockers | 0.057 | 0.28 | - | - | - | - | - | - |
| Beta-blockers | **0.18** | **<0.001** | 0.027 | 0.86 | 0.086 | 0.18 | 0.031 | 0.80 |
| Aspirins | 0.092 | 0.080 | 0.023 | 0.88 | -0.040 | 0.53 | 0.097 | 0.43 |
| Anti-microbe drugs | **0.13** | **0.013** | - | - | 0.12 | 0.055 | 0.090 | 0.46 |
| Anti-diabetic drugs | 0.080 | 0.13 | 0.089 | 0.55 | **0.14** | **0.026** | - | - |
| Diabetes diet | 0.075 | 0.16 | 0.098 | 0.51 | 0.10 | 0.10 | -0.14 | 0.27 |
| Oral medication for diabetes | **0.13** | **0.013** | 0.002 | 0.99 | 0.065 | 0.31 | 0.084 | 0.50 |
| Using insulin for diabetes | 0.035 | 0.50 | 0.11 | 0.45 | 0.12 | 0.053 | -0.12 | 0.32 |
| Anti-spasmodic drugs | 0.033 | 0.53 | - | - | 0.044 | 0.49 | - | - |
| Proton pump inhibitors | 0.090 | 0.089 | - | - | - | - | 0.15 | 0.23 |
| Anti-inflammatory drugs | -0.001 | 0.98 | -0.021 | 0.89 | 0.10 | 0.10 | 0.14 | 0.26 |
| Anti-coagulant drugs | -0.087 | 0.10 | - | - | NS | NS | - | - |
| Antidepressant drugs | 0.024 | 0.65 | -0.24 | 0.11 | -0.069 | 0.28 | - | - |
| Anxiolytic drugs | -0.092 | 0.079 | - | - | 0.030 | 0.64 | - | - |
| Insomnia drugs | 0.043 | 0.42 | -0.28 | 0.057 | -0.078 | 0.22 | - | - |
| Anti-epileptic drugs | **-0.11** | **0.039** | 0.16 | 0.29 | -0.005 | 0.94 | - | - |
| Antihistamine drugs | 0.056 | 0.29 | -0.20 | 0.17 | -0.073 | 0.26 | -0.15 | 0.24 |
| Analgesic-antipyretic drugs | 0.029 | 0.59 | 0.19 | 0.19 | 0.050 | 0.44 | 0.016 | 0.90 |
| Paracetamol usage | -0.044 | 0.41 | 0.24 | 0.11 | 0.015 | 0.81 | -0.006 | 0.96 |
| Thyroxine usage | 0.087 | 0.097 | -0.20 | 0.19 | -0.022 | 0.73 | - | - |
| Cortisone usage | 0.042 | 0.42 | - | - | 0.10 | 0.11 | - | - |
| Contraceptive pill usage | -0.096 | 0.068 | -0.15 | 0.32 | 0.066 | 0.30 | 0.062 | 0.62 |
| Female hormonal therapy | - | - | -0.067 | 0.65 | -0.067 | 0.30 | **-0.35** | **0.004** |
| Testosterone replacement | -0.079 | 0.13 | - | - | - | - | - | - |
| Multivitamin intake | -0.061 | 0.25 | 0.24 | 0.10 | -0.10 | 0.10 | -0.23 | 0.056 |
| Antioxidant intake | - | - | 0.20 | 0.19 | -0.12 | 0.052 | - | - |
| Anti-retroviral medication | - | - | - | - | - | - | - | - |
| Vertigo treatment | 0.093 | 0.077 | - | - | - | - | - | - |
| Cough syrup | - | - | - | - | **0.13** | **0.040** | -0.084 | 0.50 |
| Correlation coefficients and p-values were obtained with Spearman rank correlation analyses. Bold values denote statistical significance (p<0.050). Abbreviations: ABPM, ambulatory blood pressure monitoring; ACE, angiotensin converting enzyme; DBP, diastolic blood pressure; HDL, high-density lipoprotein; HOMA-IR, homeostatic model assessment for insulin resistance; LDL, low-density lipoprotein; MAP, mean arterial pressure; SBP, systolic blood pressure; SNS, sympathetic nervous system | | | | | | | | |

| Table S8: Spearman rank correlations between superoxide dismutase and various confounders in acute mental stress-induced adrenergic reactivity profiles (N=362) | | | | | | | | |
| --- | --- | --- | --- | --- | --- | --- | --- | --- |
| Dependent variable: Superoxide dismutase | | | | | | | | |
|  | Total population  (N=362) | | α-adrenergic  reactivity profile (n=47) | | Mixed-α/β-adrenergic  reactivity profile (n=247) | | β-adrenergic  reactivity profile (n=68) | |
|  | r | p | r | p | r | p | r | p |
| Age | -0.025 | 0.64 | -0.022 | 0.89 | -0.018 | 0.78 | -0.024 | 0.85 |
| Sex | -0.006 | 0.91 | -0.080 | 0.59 | 0.029 | 0.65 | -0.072 | 0.56 |
| Ethnicity | -0.005 | 0.92 | -0.072 | 0.63 | 0.061 | 0.34 | -0.18 | 0.14 |
| Cotinine | -0.048 | 0.37 | -0.013 | 0.93 | -0.11 | 0.10 | 0.11 | 0.39 |
| Self-reported smoking | -0.069 | 0.19 | 0.15 | 0.30 | **-0.16** | **0.014** | 0.050 | 0.69 |
| Self-reported alcohol use | 0.064 | 0.22 | 0.017 | 0.91 | 0.050 | 0.44 | 0.15 | 0.21 |
| Body mass index | -0.091 | 0.083 | -0.069 | 0.64 | -0.083 | 0.19 | -0.13 | 0.29 |
| Waist circumference | -0.094 | 0.073 | -0.13 | 0.38 | -0.076 | 0.24 | -0.12 | 0.34 |
| Cardiac output reactivity | -0.052 | 0.33 | **-0.34** | **0.019** | 0.016 | 0.80 | -0.19 | 0.13 |
| Stroke volume reactivity | -0.087 | 0.097 | **-0.31** | **0.032** | -0.007 | 0.91 | **-0.34** | **0.005** |
| Windkessel arterial compliance reactivity | 0.029 | 0.59 | -0.065 | 0.66 | 0.099 | 0.12 | -0.092 | 0.46 |
| Total peripheral resistance reactivity | 0.027 | 0.60 | 0.23 | 0.12 | -0.055 | 0.39 | 0.20 | 0.11 |
| Adrenocorticotropic hormone | 0.044 | 0.40 | 0.17 | 0.25 | 0.044 | 0.50 | -0.024 | 0.85 |
| Cortisol | 0.048 | 0.37 | 0.034 | 0.82 | 0.099 | 0.12 | -0.080 | 0.52 |
| Urinary norepinephrine-to-creatinine ratio | -0.066 | 0.21 | -0.19 | 0.21 | -0.072 | 0.26 | 0.020 | 0.87 |
| Urinary epinephrine-to-creatinine ratio | 0.072 | 0.18 | -0.056 | 0.71 | 0.036 | 0.57 | **0.28** | **0.022** |
| 24-hour ABPM SBP | -0.077 | 0.15 | 0.035 | 0.82 | **-0.14** | **0.029** | 0.033 | 0.79 |
| 24-hour ABPM DBP | -0.027 | 0.61 | 0.042 | 0.78 | -0.096 | 0.13 | 0.18 | 0.15 |
| 24-hour ABPM MAP | -0.048 | 0.36 | 0.068 | 0.65 | -0.12 | 0.054 | 0.15 | 0.22 |
| Glycated hemoglobin | -0.060 | 0.26 | 0.071 | 0.64 | -0.090 | 0.16 | -0.053 | 0.67 |
| Glucose | -0.070 | 0.18 | 0.034 | 0.82 | -0.094 | 0.14 | -0.070 | 0.57 |
| Insulin | **-0.11** | **0.042** | 0.098 | 0.51 | -0.11 | 0.080 | -0.20 | 0.10 |
| HOMA-IR | **-0.13** | **0.015** | 0.056 | 0.71 | **-0.14** | **0.031** | -0.20 | 0.097 |
| C-reactive protein | 0.018 | 0.73 | -0.13 | 0.37 | -0.008 | 0.91 | 0.17 | 0.17 |
| Tumor necrosis factor-alpha | -0.023 | 0.66 | -0.075 | 0.62 | -0.017 | 0.90 | -0.006 | 0.96 |
| Interleukin-6 | -0.013 | 0.81 | -0.24 | 0.12 | -0.016 | 0.80 | 0.11 | 0.38 |
| Total cholesterol | -0.047 | 0.37 | 0.12 | 0.44 | -0.050 | 0.43 | -0.12 | 0.33 |
| HDL-cholesterol | 0.009 | 0.87 | 0.14 | 0.34 | -0.006 | 0.93 | -0.028 | 0.82 |
| LDL-cholesterol | -0.047 | 0.38 | 0.084 | 0.57 | -0.040 | 0.53 | -0.12 | 0.32 |
| Triglycerides | **-0.11** | **0.047** | 0.028 | 0.85 | **-0.14** | **0.026** | -0.048 | 0.70 |
| Cholesterol-to-HDL ratio | -0.049 | 0.35 | -0.054 | 0.72 | -0.044 | 0.49 | -0.075 | 0.54 |
| Estimated glomerular filtration rate | -0.021 | 0.69 | -0.038 | 0.80 | 0.017 | 0.79 | -0.16 | 0.20 |
| SNS blockers | 0.069 | 0.19 | - | - | -0.12 | 0.067 | - | - |
| Statins | -0.055 | 0.30 | 0.25 | 0.092 | 0.058 | 0.36 | 0.035 | 0.77 |
| Acetylcholine inhibitors | -0.051 | 0.33 | - | - | -0.065 | 0.31 | - | - |
| ACE inhibitors | -0.046 | 0.39 | -0.24 | 0.098 | -0.029 | 0.65 | 0.018 | 0.89 |
| Angiotensin II antagonist | -0.039 | 0.46 | - | - | -0.054 | 0.40 | - | - |
| Angiotensin receptor blockers | **-0.12** | **0.028** | - | - | -0.017 | 0.79 | -0.090 | 0.46 |
| Thiazides/Diuretics | -0.079 | 0.13 | -0.051 | 0.73 | **-0.17** | **0.009** | 0.15 | 0.21 |
| Calcium-channel blockers | - | - | -0.058 | 0.70 | -0.11 | 0.099 | -0.009 | 0.94 |
| Alpha-blockers | -0.039 | 0.46 | - | - | - | - | - | - |
| Beta-blockers | -0.096 | 0.069 | -0.23 | 0.12 | 0.011 | 0.86 | -0.073 | 0.55 |
| Aspirins | -0.021 | 0.68 | -0.26 | 0.082 | 0.005 | 0.94 | -0.099 | 0.42 |
| Anti-microbe drugs | -0.045 | 0.39 | - | - | -0.049 | 0.44 | 0.050 | 0.69 |
| Anti-diabetic drugs | 0.066 | 0.21 | -0.18 | 0.23 | -0.027 | 0.68 | - | - |
| Diabetes diet | -0.016 | 0.76 | -0.087 | 0.56 | **0.13** | **0.049** | -0.18 | 0.15 |
| Oral medication for diabetes | -0.045 | 0.39 | -0.090 | 0.55 | -0.009 | 0.89 | 0.002 | 0.99 |
| Using insulin for diabetes | 0.003 | 0.96 | -0.14 | 0.37 | -0.014 | 0.83 | **-0.26** | **0.031** |
| Anti-spasmodic drugs | 0.028 | 0.60 | - | - | 0.005 | 0.94 | - | - |
| Proton pump inhibitors | 0.045 | 0.40 | - | - | - | - | 0.059 | 0.63 |
| Anti-inflammatory drugs | 0.013 | 0.81 | -0.081 | 0.59 | 0.049 | 0.45 | 0.089 | 0.47 |
| Anti-coagulant drugs | 0.027 | 0.60 | - | - | 0.020 | 0.76 | - | - |
| Antidepressant drugs | 0.018 | 0.74 | 0.011 | 0.94 | 0.038 | 0.55 | - | - |
| Anxiolytic drugs | -0.014 | 0.80 | - | - | 0.026 | 0.68 | - | - |
| Insomnia drugs | -0.028 | 0.60 | -0.016 | 0.92 | -0.030 | 0.64 | - | - |
| Anti-epileptic drugs | -0.008 | 0.87 | -0.11 | 0.47 | -0.023 | 0.72 | - | - |
| Antihistamine drugs | 0.009 | 0.87 | 0.058 | 0.70 | -0.041 | 0.53 | 0.025 | 0.84 |
| Analgesic-antipyretic drugs | -0.009 | 0.87 | -0.042 | 0.78 | -0.049 | 0.44 | 0.15 | 0.23 |
| Paracetamol usage | -0.039 | 0.46 | **0.33** | **0.025** | -0.10 | 0.12 | 0.084 | 0.49 |
| Thyroxine usage | -0.085 | 0.11 | NS | NS | -0.068 | 0.29 | - | - |
| Cortisone usage | -0.065 | 0.22 | - | - | -0.10 | 0.12 | - | - |
| Contraceptive pill usage | -0.054 | 0.30 | 0.28 | 0.057 | **-0.13** | **0.045** | -0.007 | 0.95 |
| Female hormonal therapy | - | - | -0.032 | 0.83 | -0.033 | 0.61 | -0.14 | 0.26 |
| Testosterone replacement | -0.011 | 0.84 | - | - | - | - | - | - |
| Multivitamin intake | -0.023 | 0.66 | -0.27 | 0.064 | 0.041 | 0.52 | -0.049 | 0.69 |
| Antioxidant intake | - | - | -0.21 | 0.16 | 0.015 | 0.82 | - | - |
| Anti-retroviral medication | - | - | - | - | - | - | - | - |
| Vertigo treatment | -0.056 | 0.29 | - | - | - | - | - | - |
| Cough syrup | - | - | - | - | -0.054 | 0.40 | -0.059 | 0.63 |
| Correlation coefficients and p-values were obtained with Spearman rank correlation analyses. Bold values denote statistical significance (p<0.050). Abbreviations: ABPM, ambulatory blood pressure monitoring; ACE, angiotensin converting enzyme; DBP, diastolic blood pressure; HDL, high-density lipoprotein; HOMA-IR, homeostatic model assessment for insulin resistance; LDL, low-density lipoprotein; MAP, mean arterial pressure; SBP, systolic blood pressure; SNS, sympathetic nervous system | | | | | | | | |

| Table S9: Spearman rank correlations between gamma-glutamyl transferase and various confounders in acute mental stress-induced adrenergic reactivity profiles (N=362) | | | | | | | | |
| --- | --- | --- | --- | --- | --- | --- | --- | --- |
| Dependent variable: Gamma-glutamyl transferase | | | | | | | | |
|  | Total population  (N=362) | | α-adrenergic  reactivity profile (n=47) | | Mixed-α/β-adrenergic  reactivity profile (n=247) | | β-adrenergic  reactivity profile (n=68) | |
|  | r | p | r | p | r | p | r | p |
| Age | 0.027 | 0.61 | 0.038 | 0.80 | -0.080 | 0.21 | 0.15 | 0.22 |
| Sex | **0.36** | **<0.001** | **0.53** | **<0.001** | **0.34** | **<0.001** | **0.34** | **0.005** |
| Ethnicity | **-0.56** | **<0.001** | **-0.68** | **<0.001** | **-0.50** | **<0.001** | **-0.58** | **<0.001** |
| Cotinine | **0.21** | **<0.001** | **0.33** | **0.023** | **0.17** | **0.007** | 0.19 | 0.12 |
| Self-reported smoking | **0.12** | **0.029** | **0.33** | **0.023** | 0.068 | 0.28 | 0.13 | 0.30 |
| Self-reported alcohol use | 0.006 | 0.90 | -0.17 | 0.26 | 0.051 | 0.43 | -0.063 | 0.61 |
| Body mass index | **0.33** | **<0.001** | 0.053 | 0.73 | **0.29** | **<0.001** | **0.40** | **0.001** |
| Waist circumference | **0.36** | **<0.001** | 0.26 | 0.076 | **0.35** | **<0.001** | **0.35** | **0.004** |
| Cardiac output reactivity | **-0.26** | **<0.001** | -0.17 | 0.26 | -0.091 | 0.16 | -0.20 | 0.11 |
| Stroke volume reactivity | **-0.19** | **<0.001** | 0.012 | 0.94 | -0.007 | 0.91 | 0.034 | 0.78 |
| Windkessel arterial compliance reactivity | **-0.21** | **<0.001** | **-0.29** | **0.049** | -0.10 | 0.11 | 0.028 | 0.82 |
| Total peripheral resistance reactivity | **0.29** | **<0.001** | 0.13 | 0.37 | **0.14** | **0.029** | 0.19 | 0.13 |
| Adrenocorticotropic hormone | **0.21** | **<0.001** | **0.50** | **<0.001** | **0.17** | **0.006** | 0.020 | 0.87 |
| Cortisol | 0.017 | 0.75 | **0.010** | **<0.001** | 0.044 | 0.50 | -0.18 | 0.15 |
| Urinary norepinephrine-to-creatinine ratio | -0.055 | 0.29 | **-0.073** | **<0.001** | -0.089 | 0.16 | -0.044 | 0.72 |
| Urinary epinephrine-to-creatinine ratio | -0.045 | 0.40 | 0.054 | 0.72 | -0.083 | 0.19 | 0.033 | 0.79 |
| 24-hour ABPM SBP | **0.42** | **<0.001** | **0.49** | **0.001** | **0.33** | **<0.001** | **0.57** | **<0.001** |
| 24-hour ABPM DBP | **0.48** | **<0.001** | **0.51** | **<0.001** | **0.44** | **<0.001** | **0.51** | **<0.001** |
| 24-hour ABPM MAP | **0.47** | **<0.001** | **0.52** | **<0.001** | **0.41** | **<0.001** | **0.56** | **<0.001** |
| Glycated hemoglobin | **0.42** | **<0.001** | **0.37** | **0.011** | **0.34** | **<0.001** | **0.51** | **<0.001** |
| Glucose | **0.12** | **0.027** | 0.22 | 0.15 | 0.090 | 0.16 | -0.011 | 0.93 |
| Insulin | **0.42** | **<0.001** | **0.41** | **0.004** | **0.40** | **<0.001** | **0.41** | **0.001** |
| HOMA-IR | **0.41** | **<0.001** | **0.44** | **0.002** | **0.39** | **<0.001** | **0.40** | **0.001** |
| C-reactive protein | **0.31** | **<0.001** | 0.12 | 0.43 | **0.25** | **<0.001** | **0.51** | **<0.001** |
| Tumor necrosis factor-alpha | **0.28** | **<0.001** | **0.34** | **0.019** | **0.28** | **<0.001** | 0.087 | 0.48 |
| Interleukin-6 | **0.18** | **<0.001** | 0.023 | 0.88 | **0.17** | **0.008** | 0.21 | 0.088 |
| Total cholesterol | -0.046 | 0.38 | -0.066 | 0.66 | -0.067 | 0.29 | 0.15 | 0.24 |
| HDL-cholesterol | **-0.20** | **<0.001** | -0.16 | 0.30 | **-0.22** | **0.001** | -0.13 | 0.30 |
| LDL-cholesterol | -0.047 | 0.38 | -0.088 | 0.56 | -0.067 | 0.29 | 0.15 | 0.24 |
| Triglycerides | **0.48** | **<0.001** | **0.41** | **0.004** | **0.46** | **<0.001** | **0.49** | **<0.001** |
| Cholesterol-to-HDL ratio | **0.16** | **0.003** | 0.095 | 0.52 | **0.15** | **0.019** | 0.20 | 0.11 |
| Estimated glomerular filtration rate | 0.006 | 0.91 | 0.091 | 0.54 | 0.001 | 0.98 | 0.023 | 0.85 |
| SNS blockers | 0.024 | 0.66 | - | - | 0.060 | 0.35 | - | - |
| Statins | 0.072 | 0.17 | 0.023 | 0.88 | 0.010 | 0.88 | 0.040 | 0.75 |
| Acetylcholine inhibitors | **0.12** | **0.022** | - | - | 0.081 | 0.20 | - | - |
| ACE inhibitors | 0.012 | 0.83 | 0.12 | 0.43 | 0.11 | 0.084 | 0.084 | 0.49 |
| Angiotensin II antagonist | -0.067 | 0.20 | - | - | 0.010 | 0.88 | - | - |
| Angiotensin receptor blockers | **0.16** | **0.003** | - | - | -0.048 | 0.45 | -0.084 | 0.50 |
| Thiazides/Diuretics | **0.14** | **0.009** | -0.003 | 0.99 | **0.15** | **0.016** | 0.21 | 0.088 |
| Calcium-channel blockers | - | - | 0.026 | 0.86 | **0.16** | **0.010** | 0.034 | 0.78 |
| Alpha-blockers | 0.013 | 0.80 | - | - | - | - | - | - |
| Beta-blockers | **0.24** | **<0.001** | -0.15 | 0.31 | 0.074 | 0.25 | NS | NS |
| Aspirins | 0.049 | 0.35 | -0.089 | 0.55 | 0.099 | 0.12 | 0.21 | 0.083 |
| Anti-microbe drugs | **0.14** | **0.007** | - | - | 0.12 | 0.062 | -0.050 | 0.69 |
| Anti-diabetic drugs | 0.064 | 0.23 | 0.25 | 0.092 | 0.12 | 0.062 | - | - |
| Diabetes diet | **0.15** | **0.005** | 0.11 | 0.47 | 0.079 | 0.22 | -0.18 | 0.15 |
| Oral medication for diabetes | **0.14** | **0.007** | 0.14 | 0.34 | **0.16** | **0.013** | -0.060 | 0.63 |
| Using insulin for diabetes | -0.006 | 0.92 | 0.11 | 0.47 | 0.073 | 0.25 | **-0.24** | **0.047** |
| Anti-spasmodic drugs | -0.049 | 0.35 | - | - | -0.010 | 0.87 | - | - |
| Proton pump inhibitors | 0.052 | 0.32 | - | - | - | - | -0.028 | 0.82 |
| Anti-inflammatory drugs | -0.017 | 0.74 | -0.096 | 0.52 | 0.027 | 0.68 | 0.22 | 0.076 |
| Anti-coagulant drugs | -0.058 | 0.27 | - | - | -0.023 | 0.71 | - | - |
| Antidepressant drugs | -0.036 | 0.49 | -0.17 | 0.24 | -0.071 | 0.26 | - | - |
| Anxiolytic drugs | **-0.11** | **0.034** | - | - | -0.050 | 0.43 | - | - |
| Insomnia drugs | 0.060 | 0.26 | **-0.30** | **0.041** | -0.10 | 0.12 | - | - |
| Anti-epileptic drugs | -0.083 | 0.11 | -0.11 | 0.47 | 0.10 | 0.11 | - | - |
| Antihistamine drugs | -0.055 | 0.30 | -0.12 | 0.41 | -0.050 | 0.44 | -0.15 | 0.23 |
| Analgesic-antipyretic drugs | -0.015 | 0.78 | -0.10 | 0.51 | -0.055 | 0.39 | -0.017 | 0.89 |
| Paracetamol usage | -0.018 | 0.74 | -0.016 | 0.92 | -0.035 | 0.59 | 0.073 | 0.55 |
| Thyroxine usage | 0.085 | 0.11 | 0.033 | 0.83 | -0.072 | 0.26 | - | - |
| Cortisone usage | 0.004 | 0.93 | - | - | 0.099 | 0.12 | - | - |
| Contraceptive pill usage | -0.046 | 0.39 | 0.031 | 0.84 | 0.012 | 0.86 | -0.038 | 0.76 |
| Female hormonal therapy | - | - | 0.006 | 0.97 | -0.079 | 0.22 | -0.13 | 0.30 |
| Testosterone replacement | **-0.22** | **<0.001** | - | - | - | - | - | - |
| Multivitamin intake | **-0.12** | **0.027** | -0.11 | 0.45 | **-0.26** | **<0.001** | -0.055 | 0.65 |
| Antioxidant intake | - | - | -0.22 | 0.13 | -0.11 | 0.073 | - | - |
| Anti-retroviral medication | - | - | - | - | - | - | - | - |
| Vertigo treatment | 0.044 | 0.41 | - | - | - | - | - | - |
| Cough syrup | - | - | - | - | 0.024 | 0.70 | 0.17 | 0.18 |
| Correlation coefficients and p-values were obtained with Spearman rank correlation analyses. Bold values denote statistical significance (p<0.050). Abbreviations: ABPM, ambulatory blood pressure monitoring; ACE, angiotensin converting enzyme; DBP, diastolic blood pressure; HDL, high-density lipoprotein; HOMA-IR, homeostatic model assessment for insulin resistance; LDL, low-density lipoprotein; MAP, mean arterial pressure; SBP, systolic blood pressure; SNS, sympathetic nervous system | | | | | | | | |
